# Supplementary material for: Encoding orbital angular momentum of light in space with optical catastrophes
Source: Nat Commun. 2026 Jun 8;17:7305. doi: 10.1038/s41467-026-73443-z (PMC13402805; doi:10.1038/s41467-026-73443-z)
Supplement: Supplementary file 1 — Supplementary Information [file 41467_2026_73443_MOESM1_ESM.pdf]

Supplementary Information for

Encoding orbital angular momentum of light in space with optical catastrophes

Xiaoyan Zhou[1], John You En Chan[1], Chia-Te Chang[1], Zhenchao Liu[1], Wang Hao[2,3], Andrew Forbes[4], Cheng-Wei Qiu[5, *], Hongtao Wang[1, *], & Joel K. W. Yang[1,6, *]

Affiliations

[1]Engineering Product Development, Singapore University of Technology and Design, Singapore 487372, Singapore

[2] Hangzhou International Innovation Institute, Beihang University, Hangzhou 311115, China

[3] School of Instrumentation and Optoelectronic Engineering, Beihang University, Beijing 100191 China

[4]School of Physics, University of the Witwatersrand, Private Bag 3, Wits 2050, South Africa

[5]Department of Electrical and Computer Engineering, National University of Singapore, Singapore 117583, Singapore

[6]Singapore-HUJ Alliance for Research and Enterprise (SHARE), The Smart Grippers for Soft Robotics (SGSR) Programme, Campus for Research Excellence and Technological Enterprise (CREATE), Singapore 138602, Singapore

Corresponding author

*E-mail: chengwei.qiu@nus.edu.sg

*E-mail: hongtao_wang@sutd.edu.sg

*E-mail: joel_yang@sutd.edu.sg

Inventory of Supplementary Information file:

Supplementary Note 1. Intrinsic and extrinsic orbital angular momentum.

Supplementary Note 2. OAM spectrum analysis.

Supplementary Note 3. Derivation of the angular spectrum for tailoring OAM in 3D space.

Supplementary Note 4. Derivation and analysis of the compensation phase.

Supplementary Note 5. Statistical characterization of hidden OAM.

Supplementary Note 6. Robustness of hidden OAM security to fabrication imperfections.

Supplementary Note 7. Caustic design for OAM Match.

Supplementary Note 8. Scale factor expressions for different shapes.

Supplementary Note 9. OAM Tetris.

Supplementary Note 10. Design of 3D-printed nanofins.

Supplementary Note 11. Optical setup.

Supplementary Note 1. Intrinsic and extrinsic orbital angular momentum.

Orbital angular momentum (OAM) can be classified into two distinct components: intrinsic and extrinsic. Intrinsic OAM originates from the internal phase structure of the optical wavefunction and is invariant under coordinate transformations. In contrast, extrinsic OAM arises from the spatial distribution of linear momentum relative to a chosen reference point and thus depends on the coordinate system. Disentangling these components is essential, as they stem from distinct physical origins and carry different implications. Intrinsic OAM enables robust information encoding and controlled manipulation of matter at the microscale, while extrinsic OAM may introduce coordinate-dependent artifacts in measurement. Separation between the two is therefore critical for precision applications.

The total orbital angular momentum (OAM) of a light field along the z-axis is defined as

$$J_z^{\mathrm{total}} = \left\langle xp_y - yp_x \right\rangle. \tag{1}$$

This expression can be decomposed into

$$J_z^{\mathrm{total}} = \left\langle \left(x-\langle x\rangle\right)\left(p_y-\langle p_y\rangle\right)\right\rangle - \left\langle \left(y-\langle y\rangle\right)\left(p_x-\langle p_x\rangle\right)\right\rangle + \langle x\rangle\langle p_y\rangle - \langle y\rangle\langle p_x\rangle. \tag{2}$$

The first group of terms represents the intrinsic OAM:

$$\begin{aligned} J_z^{\mathrm{int}} &= \left\langle \left(x-\langle x\rangle\right)\left(p_y-\langle p_y\rangle\right)\right\rangle - \left\langle \left(y-\langle y\rangle\right)\left(p_x-\langle p_x\rangle\right)\right\rangle \\ &= \left\langle (\mathbf{r}_\perp - \mathbf{r}_{\perp c})\times \mathbf{p}_\perp \right\rangle. \end{aligned} \tag{3}$$

The second group of terms corresponds to the extrinsic OAM:

$$\begin{aligned} J_z^{\mathrm{ext}} &= \langle x\rangle\langle p_y\rangle - \langle y\rangle\langle p_x\rangle \\ &= \mathbf{r}_{\perp c} \times \langle \mathbf{p}_\perp \rangle. \end{aligned} \tag{4}$$

Here, $\mathbf{r}_\perp = (x, y)$ is the transverse position vector. $\mathbf{r}_{\perp c}$ is the centroid. $\mathbf{p}_\perp = (p_x, p_y)$ is the transverse momentum. The symbol $\langle\cdot\rangle$ denotes the expectation operator.

To demonstrate their coordinate independence, we consider a global translation of the coordinate system: $x' = x - x_0$,and $y' = y - y_0$. Thus, $\langle x'\rangle = \langle x\rangle - x_0$, $\langle y'\rangle = \langle y\rangle - y_0$. Since momentum operators are invariant under spatial translation, the expectation values $\langle p_x\rangle$, $\langle p_y\rangle$ remain unchanged. Substituting into the expressions into the extrinsic OAM:

$$\begin{aligned} J_z^{\mathrm{ext}'} &= \langle x'\rangle\langle p_y\rangle - \langle y'\rangle\langle p_x\rangle \\ &= \langle x - x_0\rangle\langle p_y\rangle - \langle y - y_0\rangle\langle p_x\rangle. \\ &= J_z^{\mathrm{ext}} - x_0\langle p_y\rangle + y_0\langle p_x\rangle \end{aligned} \tag{5}$$

We find that the extrinsic OAM explicitly depends on the choice of coordinate origin. A shift in the reference frame alters its value, demonstrating its coordinate-dependent nature.

For the intrinsic OAM, the shifted coordinates satisfy:

$$x' - \langle x' \rangle = (x - x_0) - \langle x - x_0 \rangle = x - \langle x \rangle, \tag{6}$$

and

$$y' - \langle y' \rangle = (y - y_0) - \langle y - y_0 \rangle = y - \langle y \rangle. \tag{7}$$

The momentum and momentum expectation values remain invariant. Therefore, the intrinsic component is invariant under global translations, reflecting its coordinate-independent character.

Supplementary Note 2. OAM spectrum analysis.

The helical harmonic $e^{il\theta}$ serves as the eigenfunction of OAM and exhibits azimuthal periodicity. Based on this property, any optical beams can be expanded using helical harmonics as the basis:

$$E(r,\theta,z)=\frac{1}{\sqrt{2\pi}}\sum_{l=-\infty}^{+\infty}a_l(r,z)\exp\left(-\frac{r^2}{2\sigma^2}\right)\exp(il\theta) \tag{1}$$

with the complex coefficient

$$a_l(r,z)=\frac{1}{\sqrt{2\pi}}\int_0^{2\pi}E(r,\theta,z)\exp\left(-\frac{r^2}{2\sigma^2}\right)\exp(-il\theta)d\theta. \tag{2}$$

Here, $\exp(-r^2/2\sigma^2)$ denotes the Gaussian envelope with σ being the beam waist. Thus, the intensity of the l-th order helical harmonic is

$$C_l=\int_0^{+\infty}\left|a_l(r,z)\right|^2 rdr. \tag{3}$$

The modal power of such helical harmonics is

$$R_l=C_l/\sum_{q=-\infty}^{+\infty}C_q, \tag{4}$$

which is the OAM spectrum of light field $E(r,\theta,z)$. The OAM properties of the circular OAM beam shown in Fig. 2a are analyzed through this method of OAM spectrum decomposition. As shown in Fig. S1a, the normalized OAM spectrum reveals that the beam's energy is predominantly concentrated around a topological charge of $l=40$, which is the dominant OAM mode. To further validate the decomposition results, we reconstructed the light field by superposing the OAM components. As shown in Fig. S1b, the comparison between the original field and the reconstructed field demonstrates that the main spatial features are well preserved. The correlation coefficient between the two fields can reach 99.16% in this case, which demonstrates the reliability of the OAM spectrum decomposition method. Note that the components with weights below a threshold of 0.05 can be treated as noise-dominated contributions and are therefore excluded from the analysis to suppress the influence of experimental background.

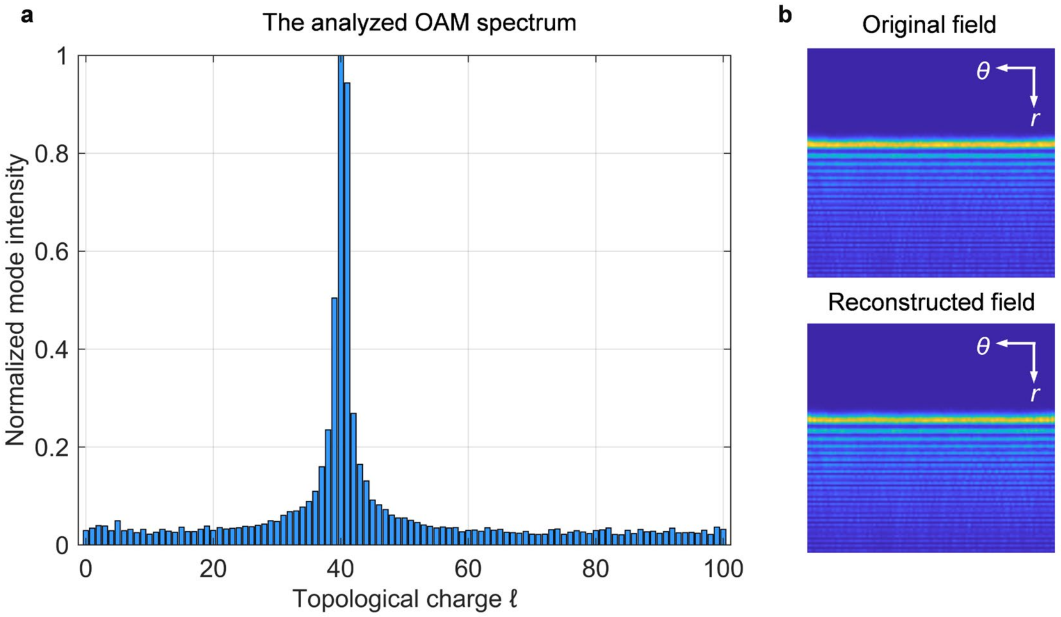

Fig. S1 OAM mode analysis and reconstruction of the OAM beam with a circular profile. a The analyzed OAM spectrum of the beam, showing the normalized mode intensity as a function of the topological charge. The energy is mainly concentrated around 40, indicating a dominant OAM mode. **b** The original field (top) and the field reconstructed by superposing the analyzed OAM modes (bottom), which are shown in polar coordinates.

Supplementary Note 3. Derivation of the angular spectrum for tailoring OAM in 3D space.
The electronic field of a beam propagating along the paraxial z direction is expressed as

$$E(x,y,z)=\exp(ikz)\iint_{-\infty}^{+\infty}F(k_x,k_y)\exp\left[i\left(k_x x+k_y y-\frac{k_x^2+k_y^2}{2k}z\right)\right]\mathrm{d}k_x\mathrm{d}k_y, \quad (1)$$

where (x,y) is an arbitrary position vector in the plane z; (k_x,k_y) is corresponding Fourier transform pair in the frequency domain; $k=2\pi/\lambda$ is the wavenumber with λ being the optical wavelength. $F(k_x,k_y)=A(k_x,k_y)\exp\left[i\Phi(k_x,k_y)\right]$ is the spectrum distribution with $A(k_x,k_y)$ and $\Phi(k_x,k_y)$ being the amplitude and the phase term, respectively. Based on the method of stationary phase, the significant contribution of the integral arises from critical points of the first kind, which satisfy

$$\begin{cases}\dfrac{\partial\Psi(k_x,k_y)}{\partial k_x}=\Phi_{k_x}(k_x,k_y)+x-\dfrac{k_x}{k}z=0\\[2ex]\dfrac{\partial\Psi(k_x,k_y)}{\partial k_y}=\Phi_{k_y}(k_x,k_y)+y-\dfrac{k_y}{k}z=0\end{cases} \quad (2)$$

with the total phase of the wave component

$$\Psi(k_x,k_y)=\Phi(k_x,k_y)+k_x x+k_y y-\frac{k_x^2+k_y^2}{2k}z. \quad (3)$$

The subscripts k_x and k_y imply the partial derivatives of the corresponding functions.

Our first step is to determine the phase $\Phi(k_x,k_y)$ in such a way that the caustics of the beams in the transverse plane is a point and enables the creation of a spatial focal curve. The parametric functions $X(z)$ and $Y(z)$ govern the trajectory of the beams with the propagation distance z acting as a parameter. Thus, we can rewrite Eq. (2) as

$$\begin{cases}\Phi_{k_x}(k_x,k_y)=\dfrac{k_x}{k}z-X(z)\\[2ex]\Phi_{k_y}(k_x,k_y)=\dfrac{k_y}{k}z-Y(z)\end{cases} \quad (4)$$

There may be many solutions to this system of equations for a particular $\tilde{z}$, and we assume that they form a continuous locus in the frequency domain, denoted as $C(\tilde{z})$. At this point, we should note that, if any wavevector (k_x,k_y) of its locus $C(\tilde{z})$ is mapped to the distance $\tilde{z}$, then a two-variable function $\tilde{z}(k_x,k_y)$ can be obtained. Moreover, it is crucial to take into account that for $\Phi(k_x,k_y)$ to be twice continuously differentiable, its mixed second-order partial derivatives should

be equal, i.e., $\Phi_{k_x k_y} = \Phi_{k_y k_x}$. Therefore, we can deduce the relationship as follows:

$$\tilde{z}_{k_y}\left(\frac{k_x}{k} - X'(\tilde{z})\right) = \tilde{z}_{k_x}\left(\frac{k_y}{k} - Y'(\tilde{z})\right), \tag{5}$$

where the subscripts k_x and k_y refer to the partial derivatives of the corresponding functions; the prime denotes differentiation with respect to $\tilde{z}$. For a certain distance $\tilde{z}$, the functions $X'(\tilde{z})$ and $Y'(\tilde{z})$ are fixed. Thus,

$$\tilde{z}_{kx}\mathrm{d}k_x + \tilde{z}_{k_y}\mathrm{d}k_y = 0\,. \tag{6}$$

By combining with Eq. (5), we obtain that

$$(k_x - kX'(\tilde{z}), k_y - kY'(\tilde{z}))\cdot(\mathrm{d}k_x, \mathrm{d}k_y) = 0. \tag{7}$$

It is clearly seen that the locus $C(\tilde{z})$ is a circle with its center at $(kX'(\tilde{z}), kY'(\tilde{z}))$, meaning that the wavevectors of the light rays that form a caustic point constitute a circle in the Fourier plane. This also aligns with the physical interpretation of the wavevector of the zero-order Bessel beams: the wavevectors $(k_\perp \cos\varphi, k_\perp \sin\varphi)$ with $k_\perp = \sqrt{k_x{}^2 + k_y{}^2} = Cons.$ and φ being the azimuth angle centered at coordinate origin $(0,0)$ constitute the caustic points at arbitrary propagation distance. From this perspective, the movement of the circle's center serves as the fundamental physical mechanism behind the curved propagation trajectory.

Now, the locus $C(\tilde{z})$ can be expressed explicitly as

$$(k_x - kX'(\tilde{z}))^2 + (k_y - kY'(\tilde{z}))^2 = \beta^2(\tilde{z}), \tag{8}$$

or equivalently,

$$k_x = kX'(\tilde{z}) + \beta(\tilde{z})\cos\varphi, \quad k_y = kY'(\tilde{z}) + \beta(\tilde{z})\sin\varphi, \tag{9}$$

where $\beta(\tilde{z})$ is the radius. Besides, it is necessary to meet the condition that the distance of the center's movement should be no greater than the variation in the circle's radius, i.e.,

$$\beta(\tilde{z}) = \beta_0 \pm nk\int_0^{\tilde{z}} \sqrt{X''(\tilde{z})^2 + Y''(\tilde{z})^2}\,\mathrm{d}\tilde{z}, \tag{10}$$

where the constant $|n| \geq 1$. Thus far, taking the partial derivative of Eq. (2) with respect to k_x (or k_y), we arrive at:

$$\frac{\partial \Psi(\tilde{z})}{\partial k_x} = \frac{k\tilde{z}_{k_x}}{2}(X'^2(\tilde{z}) + Y'^2(\tilde{z}) - \frac{\beta^2(\tilde{z})}{k^2}). \tag{11}$$

At the same time, considering the equation that

$$\frac{\partial \Psi(\tilde{z})}{\partial k_x} = \tilde{z}_{k_x} \frac{\mathrm{d}\Psi(\tilde{z})}{\mathrm{d}\tilde{z}}, \tag{12}$$

we can derive the total phase as

$$\Psi(\tilde{z}) = \frac{k}{2}\int_0^{\tilde{z}} \left[\left(\frac{\mathrm{d}X(\xi)}{\mathrm{d}\xi} \right)^2 + \left(\frac{\mathrm{d}Y(\xi)}{\mathrm{d}\xi} \right)^2 - \left(\frac{\beta(\xi)}{k} \right)^2 \right] \mathrm{d}\xi. \tag{13}$$

Here, we set the initial value to zero, since a constant phase difference does not affect the intensity distribution. Therefore, the phase distribution $\Phi(k_x, k_y)$ can be written as

$$\Phi(k_x, k_y) = \frac{k}{2}\int_0^{\tilde{z}} \left[\left(\frac{\mathrm{d}X(\xi)}{\mathrm{d}\xi} \right)^2 + \left(\frac{\mathrm{d}Y(\xi)}{\mathrm{d}\xi} \right)^2 - \left(\frac{\beta(\xi)}{k} \right)^2 \right] \mathrm{d}\xi - k_x X(\tilde{z}) - k_y Y(\tilde{z}) + \frac{k_x^2 + k_y^2}{2k}\tilde{z}, \tag{14}$$

where parameter function $\tilde{z}(k_x, k_y)$ is determined by Eq. (8). It is worth mentioning that $0 \le \beta(\tilde{z}) \le k$, which limits maximum propagation distance of the focal curve.

Going even further, we extend the caustic point in the transverse plane to a desired path $\mathbf{r}_p(\tau) = [m(\tau), n(\tau)]^{\mathrm{T}}$. The angular spectrum of the beam is represented as

$$F(k_x, k_y) = \int_0^T \exp\left[i\phi_c(\tau, \tilde{z}) + i\Phi_p(\tau, k_x, k_y) \right] \mathrm{d}\tau, \tag{15}$$

where

$$\Phi_p(\tau, k_x, k_y) = \frac{k}{2}\int_0^{\tilde{z}} \left[\left(\frac{\mathrm{d}X(\xi)}{\mathrm{d}\xi} \right)^2 + \left(\frac{\mathrm{d}Y(\xi)}{\mathrm{d}\xi} \right)^2 - \left(\frac{\beta(\xi)}{k} \right)^2 \right] \mathrm{d}\xi - k_x[X(\tilde{z}) + m(\tau)] - k_y[Y(\tilde{z}) + n(\tau)] + \frac{k_x^2 + k_y^2}{2k}\tilde{z}. \tag{16}$$

corresponds to the phase that we have derived above, which allows the trajectory of the light beam to be a curve. T is the length of the transverse path. $\phi_c(\tau, \tilde{z}) = \phi_1(\tau, \tilde{z}) + \phi_2(\tilde{z})$ represents the compensation phase to be determined, which now need be specifically expressed as a function of the length τ and the propagation distance $\tilde{z}$. By substituting Eq. (15) and Eq. (16) into Eq. (1), and considering the relationship expressed in Eq. (9), we can obtain the light field,

$$\begin{aligned} \psi(x, y, z) = \iiint & \exp\left\{ i\left[\phi_1(\tau, \tilde{z}) - kX'(\tilde{z})m(\tau) - kY'(\tilde{z})n(\tau) \right] \right\} \mathrm{d}\tau \\ & \beta(\tilde{z}) \exp\left\{ i\left[kz + \phi_2(\tilde{z}) + \Psi(\tilde{z}) + \frac{k^2X'^2(\tilde{z}) + k^2Y'^2(\tilde{z}) + \beta^2(\tilde{z})}{2k}(\tilde{z} - z) + kX'(\tilde{z})\left(x - X(\tilde{z})\right) + kY'(\tilde{z})\left(y - Y(\tilde{z})\right) \right] \right\} \mathrm{d}\tilde{z} \\ & \left[\beta'(\tilde{z}) + kX''(\tilde{z})\cos\varphi + kY''(\tilde{z})\sin\varphi \right] \exp\left\{ i\beta\left[\left(x - X(\tilde{z}) - m(\tau) - X'(\tilde{z})(z - \tilde{z})\right)\cos\varphi + \left(y - Y(\tilde{z}) - n(\tau) - Y'(\tilde{z})(z - \tilde{z})\right)\sin\varphi \right] \right\} \mathrm{d}\varphi \end{aligned} \tag{17}$$

It can be observed clearly (regarding the integration with respect to φ): the beam is composed of

the superposition of $J_0(\cdot)$, $J_1(\cdot)\cos\varphi$, $J_1(\cdot)\sin\varphi$, with its central position at $\left(X(\tilde{z})+m(\tau)+X'(\tilde{z})(z-\tilde{z}), Y(\tilde{z})+n(\tau)+Y'(\tilde{z})(z-\tilde{z})\right)$. At this point, there are two differences compared to the previous cases.

(1) The optical field is no longer the superposition of only zero-order Bessel beams, but a combination of zero-order and first-order Bessel beams.

(2) The actual superimposed components have a slight deviation from the preset center $\left(X(z)+m(\tau), Y(z)+n(\tau)\right)$. The offset is $\left(X'(\tilde{z})(z-\tilde{z})-\left(X(z)-X(\tilde{z})\right), Y'(\tilde{z})(z-\tilde{z})-\left(Y(z)-Y(\tilde{z})\right)\right)$.

The first issue can be resolved by setting $|n|\gg 1$ in Eq. (10). In this case, the light beam can be approximated as a superposition of zero-order Bessel beams. (In fact, first-order Bessel beams can also achieve similar superposition effects.) The second issue can be corrected by finding a suitable $\phi_2(\tilde{z})$. According to the stationary phase approximation, the $\tilde{z}$ that predominantly contributes to the superposition is determined by the following equation:

$$\begin{aligned}&\frac{\mathrm{d}}{\mathrm{d}\tilde{z}}\left[\phi_2(\tilde{z})+\Psi(\tilde{z})+\frac{k^2X'^2(\tilde{z})+k^2Y'^2(\tilde{z})+\beta^2(\tilde{z})}{2k}(\tilde{z}-z)+kX'\left(x-X(\tilde{z})\right)+kY'\left(y-Y(\tilde{z})\right)\right]\\&=\phi_2'(\tilde{z})+\frac{k^2X'(\tilde{z})X''(\tilde{z})+k^2Y'(\tilde{z})Y''(\tilde{z})+\beta(\tilde{z})\beta'(\tilde{z})}{k}(\tilde{z}-z)+kX''(\tilde{z})\left(x-X(\tilde{z})\right)+kY''(\tilde{z})\left(y-Y(\tilde{z})\right)=0.\end{aligned} \tag{18}$$

By adopting the least squares method, we are supposed to minimize the sum of the squared residuals between z and $\tilde{z}$, when $x=f(z)+m(\tau)$ and $y=g(z)+n(\tau)$. It can be calculated as,

$$\begin{aligned}\Delta=&\,l\phi_2'^2(\tilde{z})+2k\left(X''(\tilde{z})\int_0^T m(\tau)\mathrm{d}\tau+Y''(\tilde{z})\int_0^T n(\tau)\mathrm{d}\tau\right)\phi_2'(\tilde{z})\\&+\left(kf''(\tilde{z})\right)^2\int_0^T m^2(\tau)\mathrm{d}\tau+\left(kY''(\tilde{z})\right)^2\int_0^T n^2(\tau)\mathrm{d}\tau+k^2X''(\tilde{z})Y''(\tilde{z})\int_0^T m(\tau)n(\tau)\mathrm{d}\tau.\end{aligned} \tag{19}$$

This is a quadratic function with respect to $\phi_2'(\tilde{z})$, and it reaches its minimum value when:

$$\begin{aligned}\phi_2'(\tilde{z})&=-\frac{2k\left(X''(\tilde{z})\int_0^T m(\tau)\mathrm{d}\tau+Y''(\tilde{z})\int_0^T n(\tau)\mathrm{d}\tau\right)}{2l}\\&=-\frac{k}{l}\int_0^T\left[X''(\tilde{z})m(\tau)+Y''(\tilde{z})n(\tau)\right]\mathrm{d}\tau.\end{aligned} \tag{20}$$

By integration, we can obtain:

$$\begin{aligned}\phi_2(\tilde{z})&=-\frac{k}{T}\int_0^T\left[X'(\tilde{z})m(\tau)+Y'(\tilde{z})n(\tau)\right]\mathrm{d}\tau\\&=-k\left[X'(\tilde{z})\overline{m(\tau)}+Y'(\tilde{z})\overline{n(\tau)}\right].\end{aligned} \tag{21}$$

After the corrections, we can state that the beam now consists of a superposition of zero-order Bessel functions along the desire path. Compared with the previous example, we can derive that

$$\phi_1(\tilde{z})=\beta(\tilde{z})\tau+kX'(\tilde{z})m(\tau)+kY'(\tilde{z})n(\tau). \tag{22}$$

Therefore, the total compensation phase is given by:

$$\phi_c(\tilde{z}) = \beta(\tilde{z})\tau + kX'(\tilde{z})\left[m(\tau) - \overline{m(\tau)}\right] + kY'(\tilde{z})\left[n(\tau) - \overline{n(\tau)}\right] \quad (23)$$

In conclusion, the angular spectrum distribution for caustic beams in 3D space is given by:

$$F(\mathbf{k}_\perp) = \int_0^T \exp\left[\mathrm{i}\phi_p(\tau, \mathbf{k}_\perp) + i\phi_c(\tau)\right]\mathrm{d}\tau, \quad (24)$$

with the path phase

$$\phi_p(\tau, \mathbf{k}_\perp) = k\int_0^{\tilde{z}}\left[\mathbf{G}'^2(\xi) - \beta^2(\xi)/k^2\right]\mathrm{d}\xi/2 - \mathbf{k}_\perp\left[\mathbf{G}(\tilde{z}) + \mathbf{r}_p(\tau)\right] + k_\perp^2\tilde{z}/2k, \quad (25)$$

and compensation phase

$$\phi_c(\tau) = \beta(\tilde{z})\tau + k\mathbf{G}'(\tilde{z})[\mathbf{r}_p(\tau) - \overline{\mathbf{r}_p(\tau)}]^{\mathrm{T}}. \quad (26)$$

Here, we can define this compensation phase as $\phi_c(\tau) = \phi_{\text{length}}(\tau) + \phi_{\text{trajectory}}(\tau)$ with $\phi_{\text{length}}(\tau) = \beta(\tilde{z})\tau$ and $\phi_{\text{trajectory}}(\tau) = k\mathbf{G}'(\tilde{z})[\mathbf{r}_p(\tau) - \overline{\mathbf{r}_p(\tau)}]^{\mathrm{T}}$ for simplicity. $k = 2\pi/\lambda$ is the wavenumber with λ being the wavelength. $\mathbf{k}_\perp = (k_x, k_y)$ is the transverse wavevector. $\mathbf{G}(z) = [X(z), Y(z)]^{\mathrm{T}}$ describes the beam motion in the *x-z* and *y-z* planes respectively, in which z represents the coordinate along the optical axis. $\mathbf{G}'(\cdot)$ denotes the derivative of the function describing the propagation trajectory. $\beta^2(\tilde{z}) = \left(\mathbf{k}_\perp^{\mathrm{T}} - k\mathbf{G}'(\tilde{z})\right)^2$ is the equation of a circle in the angular spectrum, in which $\beta(\tilde{z})$ is the radius of the circle corresponding to the distance $\tilde{z}$. By mapping the wavevector (k_x, k_y) to $\tilde{z}$, the two-variable function $\tilde{z}(k_x, k_y)$ can be obtained. Actually, the center position of the circle controls the curved propagation trajectory. $\mathbf{r}_p(\tau) = [m(\tau), n(\tau)]^{\mathrm{T}}$ is the transverse caustic path and $\overline{\mathbf{r}_p(\tau)} = \int_0^T \mathbf{r}_p(\tau)\mathrm{d}\tau/T$ represents the center of the path.

Supplementary Note 4. Derivation and analysis of the compensation phase.

The derivation is carried out in a stepwise manner, starting from the simplest case of a non-diffracting beam propagating along the *z*-axis in a straight trajectory. This baseline scenario establishes the essential formulation, which is subsequently generalized to beams following a curved propagation trajectory. Within this framework, the compensation phase associated with straight-line propagation along the *z*-axis is derived from the Whittaker integral representation, with the field expressed in cylindrical coordinates,

$$\psi(\mathbf{r}) = \int_0^{2\pi} A(\varphi)\exp[i\Phi(\varphi) + ik_\perp \mathbf{r}\cdot\mathbf{u}(\varphi)]d\varphi. \tag{1}$$

Here, $\mathbf{r} = (x, y)$ denotes the transverse position vector; $\mathbf{u}(\varphi) = (\cos\varphi, \sin\varphi)$ is a unit vector specifying the propagation direction; $A(\varphi)$ and $\Phi(\varphi)$ are the amplitude and phase distributions. The caustics can be determined by evaluating the first- and second-order derivatives of the phase term in Eq. (1) with respect to φ together with the constraint relation $\mathbf{r} = (\mathbf{r}\cdot\mathbf{u})\mathbf{u} + (\mathbf{r}\cdot\mathbf{u}')\mathbf{u}'$,

$$\mathbf{r}_c(\varphi) = \frac{1}{k_\perp}[\Phi''(\varphi)\mathbf{u}(\varphi) - \Phi'(\varphi)\mathbf{u}'(\varphi)]. \tag{2}$$

Taking derivatives of both sides of Eq. (2) yields a parametric description of the caustic,

$$\mathbf{r}_c'(\varphi) = \frac{1}{k_\perp}[\Phi'''(\varphi) - \Phi'(\varphi)]\mathbf{u}(\varphi). \tag{3}$$

When the caustic degenerates into a point located at $\mathbf{r}_c(\varphi) = (x_0, y_0)$, the corresponding condition reduces to $\mathbf{r}_c'(\varphi) = 0$, i.e.,

$$\Phi'''(\varphi) - \Phi'(\varphi) = 0. \tag{4}$$

The general solution of this equation can be written as:

$$\Phi(\varphi) = A_1\cos\varphi + A_2\sin\varphi + A_3, \tag{5}$$

where A_3 has no physical significance and is therefore omitted. Consequently, Eq. (2) can be reformulated as:

$$\mathbf{r}_c(\varphi) = \frac{1}{k_\perp}(-A_1, -A_2). \tag{6}$$

The final expression of the phase function is

$$\Phi(\varphi) = -k_\perp \mathbf{r}_c(\varphi)\cdot\mathbf{u}(\varphi). \tag{7}$$

We now extend the above point-caustic solution to engineer a prescribed high-intensity path $\mathbf{r}_p(\tau)$ with τ being arc length of a parametric curve in the transverse plane. The angular spectrum associated with such a beam is expressed as

$$\begin{aligned} F(\varphi) &= A(\varphi)\exp[\mathrm{i}\Phi(\varphi)] \\ &= \int_0^T A(\tau)\exp\left[\mathrm{i}\phi_c(\tau) - \mathrm{i}k_\perp \mathbf{r}_p(\tau)\cdot\mathbf{u}(\varphi)\right]\mathrm{d}\tau, \end{aligned} \tag{8}$$

where T is the overall length of the path; $\phi_c(\tau)$ is a compensation phase that plays a crucial role in shaping the beam but remains to be determined. By applying the method of stationary phase, the dominant contributions to Eq. (8) arise from critical points of the first kind,

$$\frac{d\phi_c}{d\tau} - k_\perp \frac{d\mathbf{r}_p(\tau)}{d\tau} \cdot \mathbf{u}(\varphi) = 0, \tag{9}$$

which establishes a direct correspondence between ray-optics descriptions and caustic formation. For each position along the path parameterized by τ, a specific set of contributing rays exists, characterized by wavevectors $k_\perp \mathbf{u}(\varphi)$. These rays are required to follow the predefined trajectory in order to collectively form the desired caustic pattern. Accordingly, the associated unit vector is given by

$$\mathbf{u}(\varphi) = \frac{d\mathbf{r}_p(\tau)/d\tau}{\left|d\mathbf{r}_p(\tau)/d\tau\right|}. \tag{10}$$

The compensation phase can then be calculated as

$$\phi_c(\tau) = \phi_0 + k_\perp \int_0^\tau \left|\frac{d\mathbf{r}_p(s)}{ds}\right| ds = \phi_0 + k_\perp \tau, \tag{11}$$

where the additive constant term ϕ_0 arbitrary. For simplicity, this constant is set to zero.

On the other hand, the transverse field distribution of the beam can be written as

$$\begin{aligned} \psi(\mathbf{r}) &= \int_0^T A(\tau) \exp\left[i\phi_c(\tau)\right] \int_0^{2\pi} \exp\left[ik_\perp(\mathbf{r}_\perp - \mathbf{r}_p(\tau)) \cdot \vec{u}(\varphi)\right] d\varphi d\tau \\ &= \int_0^T A(\tau) \exp\left[i\phi_c(\tau)\right] J_0(k_\perp \left|\mathbf{r}_\perp - \mathbf{r}_p(\tau)\right|) d\tau, \end{aligned} \tag{12}$$

where $J_0(\cdot)$ denotes the zero-order Bessel function. This expression clearly indicates that the field in the initial plane consists of a superposition of zero-order Bessel functions centered along the prescribed path $\mathbf{r}_p(\tau) = [m(\tau), n(\tau)]^T$ with τ representing the arc length. To ensure the intensity is approximately uniform along the path, an appropriate weighting function $A(\tau) = 1/\sqrt{\left|\mathbf{r}_p'(\tau)\right|} = 1$ can be selected. The central challenge therefore lies in identifying a suitable form of $\phi_c(\tau)$, that maximizes the field intensity along the desired path while suppressing it in regions away from the caustic. $\phi_c(\tau) = k_\perp \int_0^\tau \left|\frac{d\mathbf{r}_p(s)}{ds}\right| ds = k_\perp \tau$ is a proper solution to this requirement.

We next consider a more general rectilinear propagation scenario in which the beam travels along a straight line that is not parallel to the *z*-axis. The propagation trajectory is parameterized as (ax, by, z), where *a* and *b* are arbitrary constants. By considering the Eq. (9) in Supplementary Note 3, the corresponding angular spectrum can be written as

$$\begin{aligned} F(\varphi) &= A(\varphi) \exp[i\Phi(\varphi)] \\ &= \int_0^T A(\tau) \exp\left[i\phi_c(\tau) - ik_\perp \mathbf{r}_p(\tau) \cdot \mathbf{u}(\varphi)\right] d\tau \\ &= \int_0^T A(\tau) \exp\left[i\phi_c(\tau) - i\left[(ak + \beta(\tilde{z})\cos\varphi) m(\tau) + (bk + \beta(\tilde{z})\sin\varphi) n(\tau)\right]\right] d\tau, \end{aligned} \tag{13}$$

where $\phi_c(\tau)$ denotes the compensation phase to be determined. The electric field at a propagation distance *z* is then evaluated using the angular spectrum method, yielding

$$\psi(x,y,z)=\exp(\mathrm{i}kz)\iint_{-\infty}^{+\infty}F(k_x,k_y)\exp\left[\mathrm{i}\left(k_x x+k_y y-\frac{k_x^2+k_y^2}{2k}z\right)\right]\mathrm{d}k_x\mathrm{d}k_y. \tag{14}$$

From this expression, one can further derive

$$\begin{aligned}\psi(x,y,z)=&\exp\left[\mathrm{i}\left(k-\frac{a^2k^2+b^2k^2+\beta^2(\tilde{z})}{2k}\right)z+\mathrm{i}\left(akx+bky\right)\right]\\&\int_0^T J_0\left(\beta(\tilde{z})\sqrt{\left(x-az-m(\tau)\right)^2+\left(y-bz-n(\tau)\right)^2}\right)\exp\left[\mathrm{i}\phi(\tau)-\mathrm{i}\left[akm(\tau)+bkn(\tau)\right]\right]\mathrm{d}\tau,\end{aligned} \tag{15}$$

which reveals that the resulting field is composed of a superposition of the zeroth-order Bessel function $J_0(\cdot)$ centered at $\left(az+m(\tau),bz+n(\tau)\right)$. In direct analogy with the straight-axis propagation case discussed earlier, the compensation phase for this tilted straight-line trajectory can be obtained as

$$\begin{aligned}\phi_c(\tau)&=\beta(\tilde{z})\int_0^\tau\left|\frac{\mathrm{d}\mathbf{r}_p(s)}{\mathrm{d}s}\right|\mathrm{d}s+akm(\tau)+bkn(\tau)\\&=\beta(\tilde{z})\tau+akm(\tau)+bkn(\tau).\end{aligned} \tag{16}$$

Building upon this result, we further generalize the propagation trajectory to an arbitrary spatial curve $(X(z),Y(z),z)$, where the functions $X(z)$ and $Y(z)$ respectively govern the trajectory of the beams in x and y directions with the propagation distance z acting as a parameter. Referring to the derivation presented in Supplementary Note 3, Eqs. (15) – (26), the compensation phase for this case can be expressed as

$$\phi_c(\tilde{z})=\ =\phi_{\text{length}}(\tau)+\phi_{\text{trajectory}}(\tau). \tag{17}$$

where $\phi_{\text{length}}(\tau)=\beta(\tilde{z})\tau$, which is determined by the length of the transverse path and $\phi_{\text{trajectory}}(\tau)=k\mathbf{G}'(\tilde{z})[\mathbf{r}_p(\tau)-\overline{\mathbf{r}_p(\tau)}]^{\mathrm{T}}$, which depends on both the propagation trajectory and transverse path on the corresponding plane. In the 2D case where the propagation trajectory is a straight line along the z-axis, $\phi_{\text{trajectory}}(\tau)=0$, so only $\phi_{\text{length}}(\tau)$ needs to be considered. In this case, Eq. (17) will accordingly be reduced to Eq. (11). However, in the more general 3D case with a curved propagation trajectory, $\phi_{\text{trajectory}}(\tau)$ is non-zero and should be taken into account in the design. To elucidate the function of the compensation phase, four distinct cases corresponding to the scenarios shown in Fig. 3 of the main text are compared in Fig. S2: the absence of compensation, the presence of ϕ_{length} or $\phi_{\text{trajectory}}$ alone, and the combined application of both phase terms. We analyze the complex-amplitude distributions in Fourier space and intensity distributions at $z=z_{\max}/5$, $z=z_{\max}/2$, and $z=4z_{\max}/5$, respectively. The results show that the target structure emerges only when both compensation phase terms are applied simultaneously.

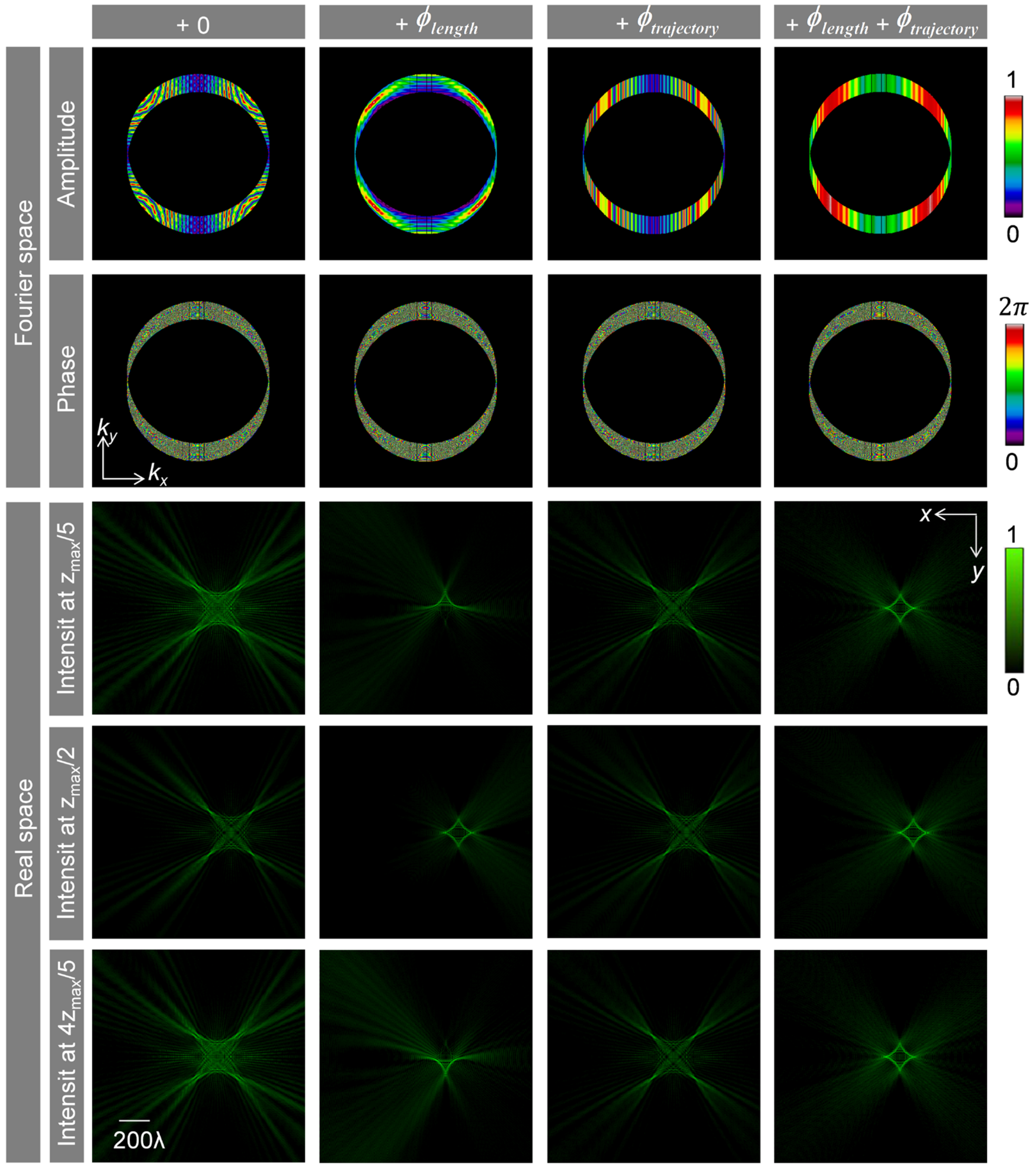

Fig. S2 Complex-amplitude distributions in Fourier space and the corresponding transverse intensity distributions in real space when different compensation phases are introduced for the case depicted in Fig. 3 of the main text. Rows 1 and 2 display the amplitude and phase distributions in Fourier space, while rows 3-5 show the transverse intensity distributions at the propagation distances $z = z_{\max}/5$, $z = z_{\max}/2$, and $z = 4z_{\max}/5$, respectively.

Supplementary Note 5. Statistical characterization of hidden OAM.

To rigorously evaluate whether the OFF-state dispersed field is statistically indistinguishable from random noise, we perform a quantitative statistical characterization of its spatial intensity distribution. Two complementary metrics are adopted: speckle contrast, which quantifies spatial intensity fluctuations, and Shannon entropy, which measures the uncertainty and information content of the intensity distribution. Together, these metrics provide a systematic assessment of structural distinguishability and statistical randomness.

1. Speckle contrast analysis

To quantitatively evaluate the spatial intensity fluctuations and detectability of the dispersed optical field in the OFF state, we analyze the speckle contrast, defined as[1]

$$C = \frac{\sigma_I}{\langle I \rangle}. \tag{1}$$

where σ_I denotes the spatial standard deviation of the intensity distribution $I(x, y)$, and $\langle I \rangle$ represents the corresponding spatial mean intensity evaluated over a selected region of interest. It should cover the main dispersed field area to ensure sufficient spatial sampling while excluding background-dominated regions. For a fully developed speckle field with stochastic phase statistics, the intensity follows a negative exponential distribution, for which the speckle contrast theoretically approaches unity.

We calculate the speckle contrast of the hidden OAM presented in the main text. In our evaluation, the background correction is performed by subtracting the dark frame intensity I_{dark}, followed by clipping negative values to zero. To suppress noise contributions and ensure sufficient signal-to-noise ratio, only pixels satisfying $I(x, y) > k\sigma_{dark}$ (with $k = 3$ in this work) are included in the statistical evaluation, where σ_{dark} denotes the standard deviation of the dark frame. As shown in Fig. S3a, the speckle contrast in the ON state is significantly larger than unity, reflecting the highly structured and non-stationary intensity distribution of the beam. In contrast, the OFF-state dispersed field yields speckle contrast values approaching unity, consistent with speckle-like randomness. This behavior indicates that the OFF-state field lacks discernible spatial structure and statistically resembles random speckle patterns rather than a structured beam.

2. Shannon entropy analysis

To further quantify statistical randomness, we evaluate the Shannon entropy of the intensity distribution[2]

$$H = -\sum_{i=1}^{N} p_i \log_2 p_i, \tag{2}$$

where p_i denotes the probability associated with the i-th intensity bin of the normalized intensity histogram. The entropy is further normalized by its maximum possible value $H_{\max} = \log_2 N$ to enable direct comparison between different states. The normalized intensity

histogram here is constructed using $N = 256$ bins, corresponding to an 8-bit discretization of the intensity range. As shown in Fig. S3b, the OFF-state dispersed field exhibits substantially higher normalized Shannon entropy than the ON-state structured beam and approaches the entropy level of numerically generated random phase fields. This increase in entropy reflects a broader and more uniform intensity probability distribution, indicating reduced predictability and diminished structural information content.

The combined results of speckle contrast (approaching unity) and high normalized Shannon entropy demonstrate that the OFF-state field exhibits statistical properties consistent with a random speckle field. It implies that the hidden OAM information is statistically obfuscated at the physical layer, significantly reducing the feasibility of computational decryption or reconstruction.

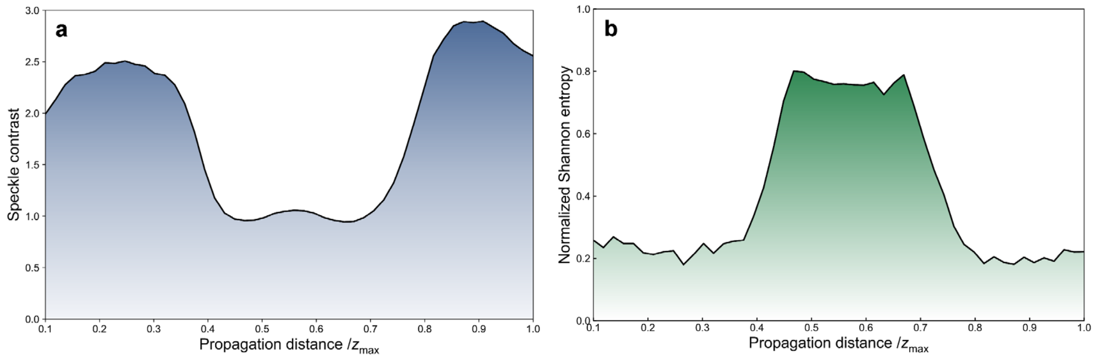

Fig. S3 Statistical characterization of the hidden OAM field. Speckle contrast (a) and normalized Shannon entropy (b) as a function of propagation distance.

Supplementary Note 6. Robustness of hidden OAM security to fabrication imperfections. The fabrication imperfections inherent to the TPL process can be regarded as randomly distributed geometric perturbations. These perturbations effectively reflect realistic structural deviations, including dimensional variations and rounding effects arising from the finite focal voxel. Here, we investigate their influence on the hidden OAM field. Random distribution functions f_A and f_ϕ are introduced to characterize the amplitude and phase perturbations, respectively. The corresponding random factors a and b quantify their fluctuation magnitudes. Accordingly, the perturbed amplitude and phase are expressed as $A^{'} = A_0\left[1+af_A\right]$ and $\phi^{'} = \phi_0\left[1+af_\phi\right]$, where A_0 and ϕ_0 denote the ideal initial amplitude and phase distributions of the hidden OAM field.

In the robustness analysis, the random factors are varied within the range of 0–0.1, corresponding to up to 10% perturbations in amplitude and phase. This interval is selected to approximate realistic fabrication tolerances in two-photon lithography. As shown in Fig. S4, the dispersed hidden OAM field in the OFF state exhibits a speckle contrast close to unity, while the normalized Shannon entropy remains within the range of approximately 0.7–0.8. These characteristics indicate that the OFF-state field is statistically indistinguishable from random noise, reflecting a high degree of statistical randomness. In contrast, in the ON state the structured OAM pattern is constructed within the designated propagation distances. The speckle contrast deviates from unity following the characteristic evolution, accompanied by a reduction in normalized Shannon entropy relative to the OFF state. Although higher perturbation levels introduce moderate spatial blurring, the statistical separation between the OFF (noise-like, high-entropy) and ON (structured, lower-entropy) regimes remains clearly preserved, ensuring reliable discrimination and robust security performance.

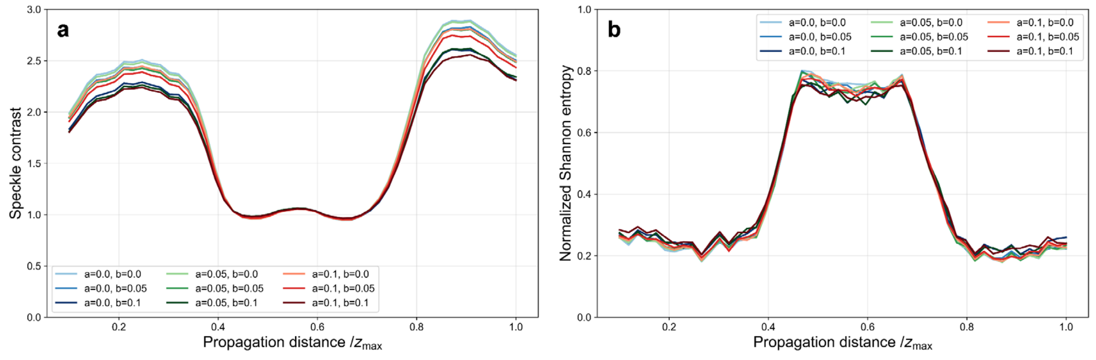

Fig. S4 Statistical characterization of the hidden OAM field under fabrication imperfections. Speckle contrast (a) and normalized Shannon entropy (b) as functions of propagation distance for varying levels of fabrication imperfections.

Supplementary Note 7. Caustic design for OAM Match.

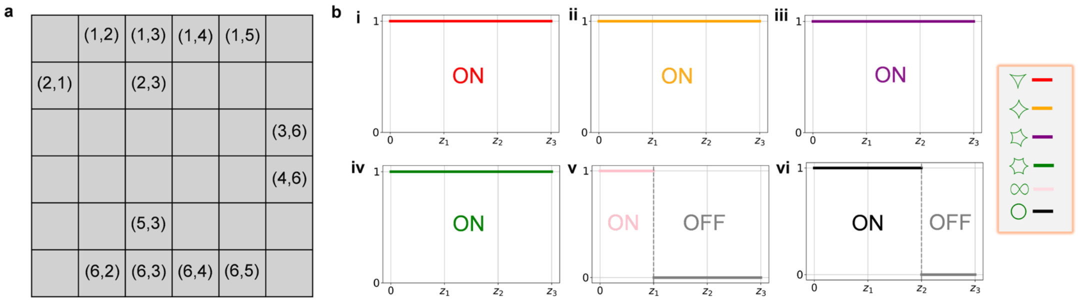

Fig. S5 Working principle of OAM Match based on caustic design. a The coordinate information, which is used to locate the positions of the caustic switches in different channels. **b** The design equation for caustic switches across different channels, with binary values 1 and 0 respectively indicating the ON and OFF states. The switch configurations are defined as follows: b(i) for channels (1,2) and (2,1); b(ii) for (3,6) and (6,3); b(iii) for (1,4), (4,6), and (6,3); b(iv) for (1,3) and (6,5); b(v) for (1,5) and (5,3); and b(vi) for (2,3) and (6,2).

Supplementary Note 8. Scale factor expressions for different shapes.

The scale factor is denoted by *a*, which characterizes the overall size of the pattern. For all parametric equations, the parameter t is defined in the range $t \in (0, 2\pi]$.

Circle:

$$x(t) = a\cos t, \qquad y(t) = a\sin t.$$

Lemniscate of Bernoulli:

$$x(t) = \frac{\sqrt{2}a\cos t}{1+\sin^2 t}, \qquad y(t) = \frac{\sqrt{2}a\sin t\cos t}{1+\sin^2 t}.$$

n-cusped hypocycloid:

$$x(t) = (a - a/n)\cos t + a/n\cos(\frac{a - a/n}{a/n}t),$$
$$y(t) = (a - a/n)\sin t - a/n\sin(\frac{a - a/n}{a/n}t).$$

Supplementary Note 9. OAM Tetris.

The design principle of the OAM Tetris is illustrated in Fig. S6a. The target block configuration (first column) is defined by two key elements: the intensity profiles (second column) and their associated average OAM (third column, visually distinguished by different background colors). Each group consists of four adjacent blocks exhibiting identical intensity profiles and average OAM. The details for OAM switch on each channel are presented in Fig. S7.

The propagation dynamics of the OAM Tetris are shown in Fig. S6b. At the propagation distance z_1, four distinct Tetris block groups emerge, each exhibiting characteristic caustic patterns: an O-shaped group with a deltoid caustic, an L-shaped group with an astroid caustic, an I-shaped group with a five-cusped caustic, and a T-shaped group with a six-cusped caustic. The corresponding average OAM are 12, 3, 9, and 6, respectively. As the propagation advances to z_2, the T-shaped group fills the gaps at the bottom, completing the lower two rows. At z_3, these two rows disappear, and the remaining blocks shift downward. Simultaneously, a new T-shaped group with an average OAM of 6 emerges, exhibiting the deltoid-shaped pattern. These dynamics are consistent with the fundamental rules of the Tetris game.

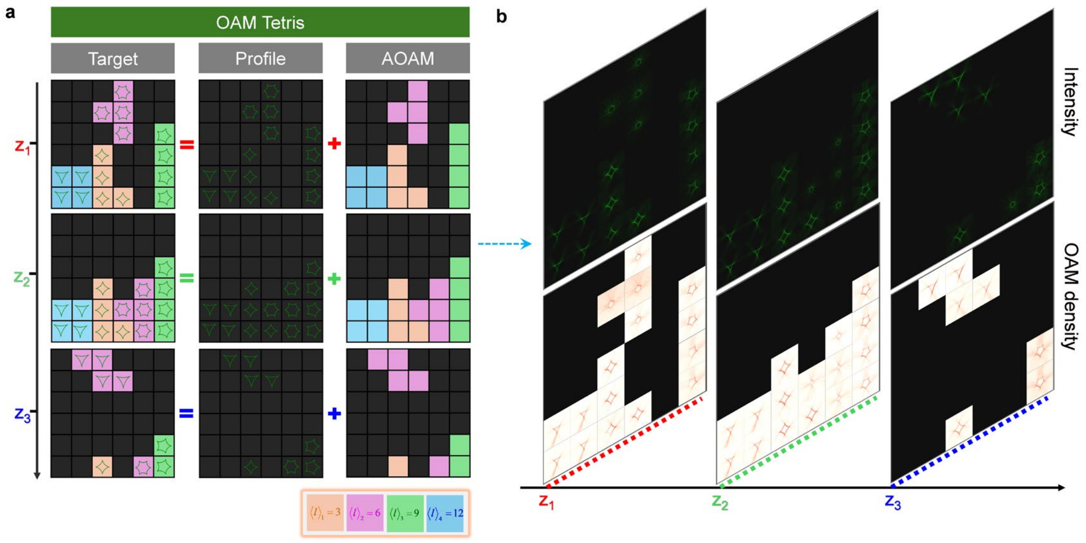

Fig. S6 OAM switch array inspired by 'Tetris'-style puzzle games. a Schematic illustration of a 6 × 6 OAM beam array. The first column shows the target OAM beams at propagation distances z_1, z_2, and z_3, which are tailored through both the intensity profiles in the second column and the average OAM in the third column. **b** Simulated transverse intensity patterns and OAM density distributions of the generated beams at three observation planes. AOAM, average OAM.

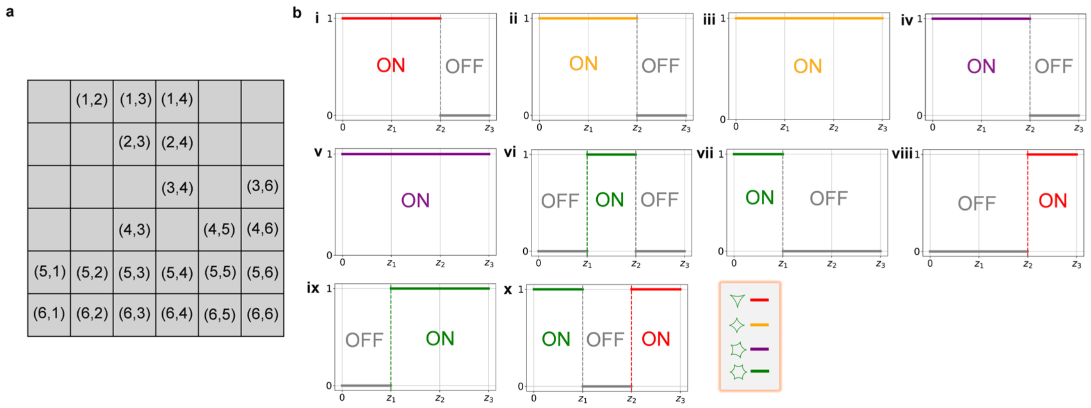

Fig. S7 Working principle of OAM Tetris based on caustic design. a The coordinate information, which is used to locate the positions of the caustic switches in different channels. **b** The design equation for caustic switches across different channels, with binary values 1and 0 respectively indicating the ON and OFF states. The switch configurations are defined as follows: b(i) for channels (5,1), (5,2), (6,1) and (6,2); b(ii) for (4,3), (5,3) and (6,4); b(iii) for (6,3); b(iv) for (3,6) and (4,6); b(v) for (5,6) and (6,6); and b(vi) for (4,5), (5,4) and (5,5); b(vii) for (1,4) and (3,4); b(viii) for (1,2) and (1,3); b(ix) for (6,5); b(x) for (2,3) and (2,4).

Supplementary Note 10. Design of 3D-printed nanofins.

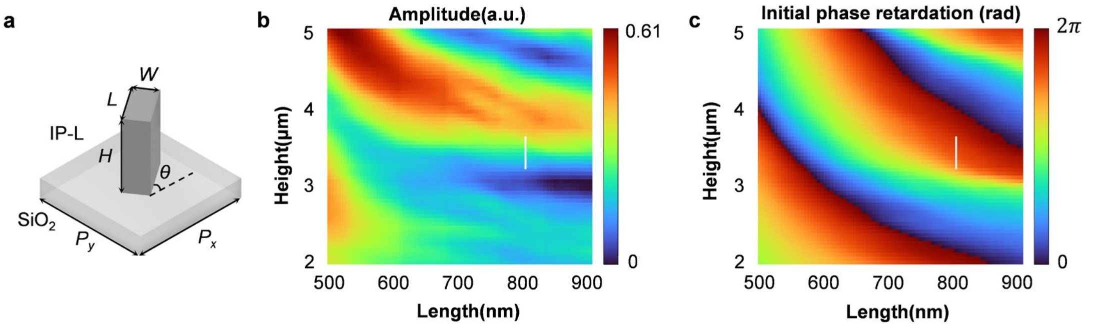

Fig. S8 Design and optimization of 3D-printed nanofins for control of complex-amplitude responses in transmitted light. a Schematic of subwavelength IP-L nanofins on SiO_2 substrate. θ indicates the in-plane rotation angle of IP-L nanofins. **b, c** Numerical characterization of cross-polarization amplitude (**b**) and initial phase retardation (**c**) after transmission through nanopillars of different heights and widths. Selected nanofins are marked with white line in the figures.

Supplementary Note 11. Optical setup.

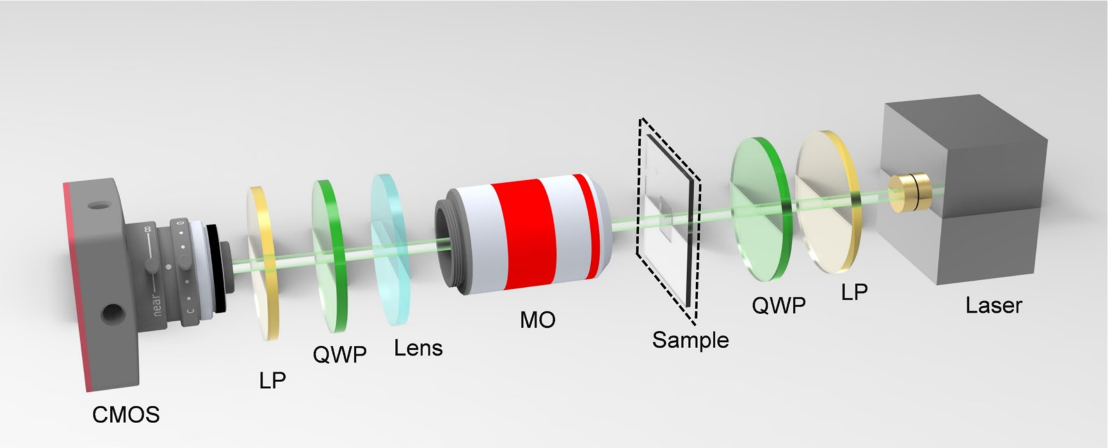

Fig. S9 Schematic diagram of the optical setup. LP linear polarizer, QWP quarter-wave plate, MO microscope objective; CMOS complementary metal oxide semiconductor.

References

[1] Goodman, J.W. *Statistical optics*, (John Wiley & Sons, 2015).

[2] Cover, T.M. *Elements of information theory*, (John Wiley & Sons, 1999).
