# Supplementary material for: Encoding orbital angular momentum of light in space with optical catastrophes
Source: Nat Commun. 2026 Jun 8;17:7305. doi: 10.1038/s41467-026-73443-z (PMC13402805; doi:10.1038/s41467-026-73443-z)
Supplement: Supplementary file 2 — Description of Additional Supplementary Files [file 41467_2026_73443_MOESM2_ESM.pdf]

Description of Additional Supplementary Files

Supplementary Movie: Fabrication process
