# Supplementary material for: Encoding orbital angular momentum of light in space with optical catastrophes
Source: Nat Commun. 2026 Jun 8;17:7305. doi: 10.1038/s41467-026-73443-z (PMC13402805; doi:10.1038/s41467-026-73443-z)
Supplement: Supplementary file 4 — Transparent Peer Review file [file 41467_2026_73443_MOESM4_ESM.pdf]

nature portfolio

Peer Review File

Encoding orbital angular momentum of light in space with optical catastrophes

Corresponding Author: Professor Joel Yang

This file contains all reviewer reports in order by version, followed by all author rebuttals in order by version.

Version 0:

Reviewer comments:

Reviewer #1

(Remarks to the Author)
In this manuscript, the authors propose and experimentally demonstrate an approach to tailoring the structure and dynamic evolution of optical vortex (OV) beams. They use caustics method and optical catastrophes theory to achieve both longitudinal and transverse shaping of light, including tailoring intrinsic orbital angular momentum (OAM), customizing OAM variants, and the ‘OAM hidden’ phenomenon. They achieve such versatile light tailoring based on metasurfaces which are manufactured by performing two-photon polymerization lithography on indium tin oxide-coated glass substrates. Furthermore, they demonstrate that the proposed technique can be utilized in encryption applications—high-dimensional encryption demos (with the principle analogous to the rule of the game Candy Crush and Tetris). To my point of view, the idea is interesting. The authors may consider the following comments before its publication in Nature Communications.
1. The authors should provide more details on the two phase terms for caustic compensation, i.e., ϕ_length and ϕ_trajectory.
2. The ‘OAM hidden’ phenomenon is quite interesting. What’s the in-depth physical mechanism to achieve this phenomenon? Maybe destructive interference?
3. For encryption applications, in order to achieve high security, ciphertexts and local keys are required. In the proof-of-concept demonstration, what are the ciphertexts and local keys of the decryption process?
4. Why did the authors characterize the average OAM value based on observing the size of intensity profiles of generated beam? Also, the authors declare that they use the OAM decomposition principle, which calculate the overlap between the light field and ideal OV modes, to calculate the OAM spectrum to obtain average OAM value. So how to measure the complex wavefront of generated light field to calculate its OAM spectrum in experiment? The authors may add some discussions and perspective on it.
5. The authors declare that their technique features an information capacity of I=log_2 A×B×M×P^K bits. However, there are no descriptions on the practical bound of it. For instance, how does the crosstalk between adjacent "OAM beam units" in the array limit the A×B term? What are the realistic limits for the distinguishable OAM orders (M) and beam profile classes (P)?

Reviewer #2

(Remarks to the Author)
This paper presents an innovative implementation of orbital angular momentum (OAM) sculpting in 3D space based on the optical catastrophe theory and complex-amplitude metasurfaces fabricated by TPL. The authors have demonstrated an capability to manipulate OAM profiles along pre-designed spatial trajectories. Very interestingly, they introduced a novel concept of hidden OAM to physically conceal information during propagation and reconstruct it at specific spatial coordinates for the application of enhanced optical security. In my opinion, this introduces a new concept of encryption that potentially surpasses traditional multiplexing methods.

Nevertheless, considering the authors’ previous work on spatial caustics and the maturity of OAM research, the manuscript in its current form requires further supporting analysis to warrant publication in Nature Communications. Specifically, to emphasize the novelty rather than the incremental advances, I think it is essential to rigorously analyze the applicability of encryption and anti-eavesdropping features based on the caustic OAM.

1. Could the authors perform the comparative analysis of the presented work with the previous OAM sculpting to clearly show the main novelty of intrinsic OAM? For example, in the author’s previous work (Nat. Communi. 2024, 15, 3719), the

roatated energy flow of caustic beams also exhibited OAM. How different the purity of intrinsic component (99%) of presented work with other works including the author's previous work?

2. Considering the author’s previous works on caustic sculpting, I think the concept of hidden OAM is one of the most intriguing novelty of this study for the applications of anti-eavesdropping. However, the claim that the beam is hidden simply because the intensity drops by 2 orders of magnitude is qualitative. To validate this as a robust security applications, would the author quantify the randomness of the dispersed field in the OFF state? For example, evaluation of speckle contrast (or shannon entropy if possible) could statistically prove that the information is indistinguishable from random noise and safe from recently developed computational decryption.

3. Related, could the authors show the tolerance of the fabrication errors by TPL process to the hidden OAM security?

4. The authors demonstrate the encryption scheme using a 6 by 6 array. However, given that the period of the metasurface is larger than the wavelength, coherent diffraction such as the Talbot effect will be inevitably significant for the claimed high-capacity applications involving larger arrays (e.g., 10 by 10 or larger), which could degrade the signal by overlapping with the hidden or active OAM of neighboring region. Could the author discuss and analyze the relationship between the key parameters including pixel pitch size, propagation and capacity limit for the potential high capacity applications.

Minor points.

5. In the spectrum analysis, components below a threshold of 0.05 were removed to calculate the OAM purity. Would the authors justify this specific threshold value? A brief sensitivity analysis about the calculated purity versus different thresholds (e.g., 0.01 vs 0.1) would confirm that the reported high purity is stable and robust.

6. In Figure 5d, the scale factor serves as the lookup key to retrieve the average OAM value for decryption. While it intuitively implies the size of the pattern, a explicit definition is missing.

Version 1:

Reviewer comments:

Reviewer #1

(Remarks to the Author)
The authors have addressed all of my comments. Thus, I'm pleased to recommend its publication in Nature Communications.

Reviewer #2

(Remarks to the Author)
The authors have well addressed my comments, and the revised manuscript clearly demonstrates strong novelty and significance.
I therefore recommend this manuscript for the publication in Nature Communications.

Open Access This Peer Review File is licensed under a Creative Commons Attribution 4.0 International License, which permits use, sharing, adaptation, distribution and reproduction in any medium or format, as long as you give appropriate credit to the original author(s) and the source, provide a link to the Creative Commons license, and indicate if changes were made.
In cases where reviewers are anonymous, credit should be given to 'Anonymous Referee' and the source.
The images or other third party material in this Peer Review File are included in the article's Creative Commons license, unless indicated otherwise in a credit line to the material. If material is not included in the article's Creative Commons license and your intended use is not permitted by statutory regulation or exceeds the permitted use, you will need to obtain permission directly from the copyright holder.
To view a copy of this license, visit https://creativecommons.org/licenses/by/4.0/

Reply to reviewers and changes made accordingly:

Reviewer 1:

"In this manuscript, the authors propose and experimentally demonstrate an approach to tailoring the structure and dynamic evolution of optical vortex (OV) beams. They use caustics method and optical catastrophes theory to achieve both longitudinal and transverse shaping of light, including tailoring intrinsic orbital angular momentum (OAM), customizing OAM variants, and the 'OAM hidden' phenomenon. They achieve such versatile light tailoring based on metasurfaces which are manufactured by performing two-photon polymerization lithography on indium tin oxide-coated glass substrates. Furthermore, they demonstrate that the proposed technique can be utilized in encryption applications—high-dimensional encryption demos (with the principle analogous to the rule of the game Candy Crush and Tetris). To my point of view, the idea is interesting. The authors may consider the following comments before its publication in Nature Communications."

We thank the reviewer for the positive and encouraging assessment of our work. The constructive comments are addressed point by point below.

Comment 1: The authors should provide more details on the two phase terms for caustic compensation, i.e., ϕ_length and ϕ_trajectory.

Reply: We thank the reviewer for this constructive suggestion. In the revised manuscript, we have added a dedicated discussion of the two compensation phase terms in Supplementary Note 4. This section includes a detailed derivation of the compensation phase, as well as an explanation of the importance of these two components in achieving the desired field profiles along the prescribed trajectories. In addition, a supplementary figure is presented to visually illustrate the resulting intensity distributions in the absence of compensation, as well as when each compensation phase term is applied individually and in combination. We have also provided an explanation in the revised main text, as follows:

…Across these three selected planes, the caustic points are arranged along an astroid path and sequentially superposed following the rainbow colors. To ensure the transverse wavevectors remain tangential to the path and the constructive interference occurs at every caustic point, the compensation phase, $\phi_{\text{length}}(\tau)+\phi_{\text{trajectory}}(\tau)$, is applied throughout the process (details in Supplementary Note 4).

Supplementary Note 4. Derivation and analysis of the compensation phase.

The derivation is carried out in a stepwise manner, starting from the simplest case of a non-diffracting beam propagating along the *z*-axis in a straight trajectory. This baseline scenario establishes the essential formulation, which is subsequently generalized to beams following a curved propagation trajectory. Within this framework, the compensation phase associated with straight-line propagation along the *z*-axis is derived from the Whittaker integral representation, with the field expressed in cylindrical coordinates,

$$\psi(\mathbf{r}) = \int_0^{2\pi} A(\varphi)\exp[i\Phi(\varphi) + ik_\perp \mathbf{r}\cdot\mathbf{u}(\varphi)]d\varphi. \tag{1}$$

Here $\mathbf{r} = (x, y)$ denotes the transverse position vector; $\mathbf{u}(\varphi) = (\cos\varphi, \sin\varphi)$ is a unit vector specifying the propagation direction; $A(\varphi)$ and $\Phi(\varphi)$ are the amplitude and phase distributions. The caustics can be determined by evaluating the first- and second-order derivatives of the phase term in Eq. (1) with respect to φ together with the constraint relation $\mathbf{r} = (\mathbf{r}\cdot\mathbf{u})\mathbf{u} + (\mathbf{r}\cdot\mathbf{u}')\mathbf{u}'$,

$$\mathbf{r}_c(\varphi) = \frac{1}{k_\perp}[\Phi''(\varphi)\mathbf{u}(\varphi) - \Phi'(\varphi)\mathbf{u}'(\varphi)]. \tag{2}$$

Taking derivatives of both sides of Eq. (2) yields a parametric description of the caustic,

$$\mathbf{r}_c'(\varphi) = \frac{1}{k_\perp}[\Phi'''(\varphi) - \Phi'(\varphi)]\mathbf{u}(\varphi). \tag{3}$$

When the caustic degenerates into a point located at $\mathbf{r}_c(\varphi) = (x_0, y_0)$, the corresponding condition reduces to $\mathbf{r}_c'(\varphi) = 0$, i.e.,

$$\Phi'''(\varphi) - \Phi'(\varphi) = 0. \tag{4}$$

The general solution of this equation can be written as:

$$\Phi(\varphi) = A_1\cos\varphi + A_2\sin\varphi + A_3, \tag{5}$$

where A_3 has no physical significance and is therefore omitted. Consequently, Eq. (2) can be reformulated as:

$$\mathbf{r}_c(\varphi) = \frac{1}{k_\perp}(-A_1, -A_2). \tag{6}$$

The final expression of the phase function is

$$\Phi(\varphi) = -k_\perp \mathbf{r}_c(\varphi)\cdot\mathbf{u}(\varphi). \tag{7}$$

We now extend the above point-caustic solution to engineer a prescribed high-intensity path $\mathbf{r}_p(\tau)$ with τ being arc length of a parametric curve in the transverse plane. The angular spectrum associated with such a beam is expressed as

$$\begin{aligned} F(\varphi) &= A(\varphi)\exp[\mathrm{i}\Phi(\varphi)] \\ &= \int_0^T A(\tau)\exp\left[\mathrm{i}\phi_c(\tau) - \mathrm{i}k_\perp \mathbf{r}_p(\tau)\cdot\mathbf{u}(\varphi)\right]\mathrm{d}\tau, \end{aligned} \tag{8}$$

where T is the overall length of the path; $\phi_c(\tau)$ is a compensation phase that plays a crucial role in shaping the beam but remains to be determined. By applying the method of stationary phase, the dominant contributions to Eq. (8) arise from critical points of the first kind,

$$\frac{\mathrm{d}\phi_c}{\mathrm{d}\tau} - k_\perp \frac{\mathrm{d}\mathbf{r}_p(\tau)}{\mathrm{d}\tau}\cdot\mathbf{u}(\varphi) = 0, \tag{9}$$

which establishes a direct correspondence between ray-optics descriptions and caustic formation. For each position along the path parameterized by τ, a specific set of contributing rays exists, characterized by wavevectors $k_\perp \mathbf{u}(\varphi)$. These rays are required to follow the predefined trajectory in order to collectively form the desired caustic pattern. Accordingly, the associated unit vector is given by

$$\mathbf{u}(\varphi) = \frac{\mathrm{d}\mathbf{r}_p(\tau)/d\tau}{\left|\mathrm{d}\mathbf{r}_p(\tau)/d\tau\right|}. \tag{10}$$

The compensation phase can then be calculated as

$$\phi_c(\tau)=\phi_0+k_\perp\int_0^\tau\left|\frac{\mathrm{d}\mathbf{r}_p(s)}{\mathrm{d}s}\right|\mathrm{d}s=\phi_0+k_\perp\tau, \tag{11}$$

where the additive constant term ϕ_0 arbitrary. For simplicity, this constant is set to zero.

On the other hand, the transverse field distribution of the beam can be written as

$$\begin{aligned}\psi(\mathbf{r})&=\int_0^T A(\tau)\exp\left[\mathrm{i}\phi_c(\tau)\right]\int_0^{2\pi}\exp\left[\mathrm{i}k_\perp(\mathbf{r}_\perp-\mathbf{r}_p(\tau))\cdot\vec{u}(\varphi)\right]\mathrm{d}\varphi\mathrm{d}\tau\\&=\int_0^T A(\tau)\exp\left[\mathrm{i}\phi_c(\tau)\right]J_0(k_\perp\left|\mathbf{r}_\perp-\mathbf{r}_p(\tau)\right|)\mathrm{d}\tau,\end{aligned} \tag{12}$$

where $J_0(\cdot)$ denotes the zero-order Bessel function. This expression clearly indicates that the field in the initial plane consists of a superposition of zero-order Bessel functions centered along the prescribed path $\mathbf{r}_p(\tau)=[m(\tau),n(\tau)]^{\mathrm{T}}$ with τ representing the arc length. To ensure the intensity is approximately uniform along the path, an appropriate weighting function $A(\tau)=1/\sqrt{\left|\mathbf{r}_p'(\tau)\right|}=1$ can be selected. The central challenge therefore lies in identifying a suitable form of $\phi_c(\tau)$, that maximizes the field intensity along the desired path while suppressing it in regions away from the caustic. $\phi_c(\tau)=k_\perp\int_0^\tau\left|\frac{\mathrm{d}\mathbf{r}_p(s)}{\mathrm{d}s}\right|\mathrm{d}s=k_\perp\tau$ is a proper solution to this requirement.

We next consider a more general rectilinear propagation scenario in which the beam travels along a straight line that is not parallel to the z-axis. The propagation trajectory is parameterized as (ax,by,z), where a and b are arbitrary constants. By considering the Eq. (9) in Supplementary Note 3, the corresponding angular spectrum can be written as

$$\begin{aligned}F(\varphi)&=A(\varphi)\exp[\mathrm{i}\Phi(\varphi)]\\&=\int_0^T A(\tau)\exp\left[\mathrm{i}\phi_c(\tau)-\mathrm{i}k_\perp\mathbf{r}_p(\tau)\cdot\mathbf{u}(\varphi)\right]\mathrm{d}\tau\\&=\int_0^T A(\tau)\exp\left[\mathrm{i}\phi_c(\tau)-\mathrm{i}\left[(ak+\beta(\tilde{z})\cos\varphi)m(\tau)+(bk+\beta(\tilde{z})\sin\varphi)n(\tau)\right]\right]\mathrm{d}\tau,\end{aligned} \tag{13}$$

where $\phi_c(\tau)$ denotes the compensation phase to be determined. The electric field at a propagation distance z is then evaluated using the angular spectrum method, yielding

$$\psi(x,y,z)=\exp(\mathrm{i}kz)\iint_{-\infty}^{+\infty}F(k_x,k_y)\exp\left[\mathrm{i}\left(k_x x+k_y y-\frac{k_x^2+k_y^2}{2k}z\right)\right]\mathrm{d}k_x\mathrm{d}k_y. \tag{14}$$

From this expression, one can further derive

$$\begin{aligned}\psi(x,y,z)&=\exp\left[\mathrm{i}\left(k-\frac{a^2k^2+b^2k^2+\beta^2(\tilde{z})}{2k}\right)z+\mathrm{i}\left(akx+bky\right)\right]\\&\int_0^T J_0\left(\beta(\tilde{z})\sqrt{\left(x-az-m(\tau)\right)^2+\left(y-bz-n(\tau)\right)^2}\right)\exp\left[\mathrm{i}\phi(\tau)-\mathrm{i}\left[akm(\tau)+bkn(\tau)\right]\right]\mathrm{d}\tau,\end{aligned} \tag{15}$$

which reveals that the resulting field is composed of a superposition of the zeroth-order Bessel function $J_0(\cdot)$ centered at $\left(az+m(\tau),bz+n(\tau)\right)$. In direct analogy with the straight-axis propagation case discussed earlier, the compensation phase for this tilted straight-line trajectory can be obtained as

$$
\begin{aligned}
\phi_c(\tau) &= \beta(\tilde{z})\int_0^{\tau}\left|\frac{\mathrm{d}\mathbf{r}_p(s)}{\mathrm{d}s}\right|\mathrm{d}s + akm(\tau) + bkn(\tau) \\
&= \beta(\tilde{z})\tau + akm(\tau) + bkn(\tau).
\end{aligned} \tag{16}
$$

Building upon this result, we further generalize the propagation trajectory to an arbitrary spatial curve $(X(z), Y(z), z)$, where the functions $X(z)$ and $Y(z)$ respectively govern the trajectory of the beams in x and y directions with the propagation distance z acting as a parameter. Referring to the derivation presented in Supplementary Note 3, Eqs. (15) – (26), the compensation phase for this case can be expressed as

$$
\phi_c(\tilde{z}) = \; = \phi_{\text{length}}(\tau) + \phi_{\text{trajectory}}(\tau). \tag{17}
$$

where $\phi_{\text{length}}(\tau) = \beta(\tilde{z})\tau$, which is determined by the length of the transverse path and $\phi_{\text{trajectory}}(\tau) = k\mathbf{G}'(\tilde{z})[\mathbf{r}_p(\tau) - \overline{\mathbf{r}_p(\tau)}]^{\mathrm{T}}$, which depends on both the propagation trajectory and transverse path on the corresponding plane. In the 2D case where the propagation trajectory is a straight line along the z-axis, $\phi_{\text{trajectory}}(\tau) = 0$, so only $\phi_{\text{length}}(\tau)$ needs to be considered. In this case, Eq. (17) will accordingly be reduced to Eq. (11). However, in the more general 3D case with a curved propagation trajectory, $\phi_{\text{trajectory}}(\tau)$ is non-zero and should be taken into account in the design. To elucidate the function of the compensation phase, four distinct cases corresponding to the scenarios shown in Fig. 3 of the main text are compared in Fig. S2: the absence of compensation, the presence of ϕ_{length} or $\phi_{\text{trajectory}}$ alone, and the combined application of both phase terms. We analyze the complex-amplitude distributions in Fourier space and intensity distributions at $z = z_{\max}/5$, $z = z_{\max}/2$, and $z = 4z_{\max}/5$, respectively. The results show that the target structure emerges only when both compensation phase terms are applied simultaneously.

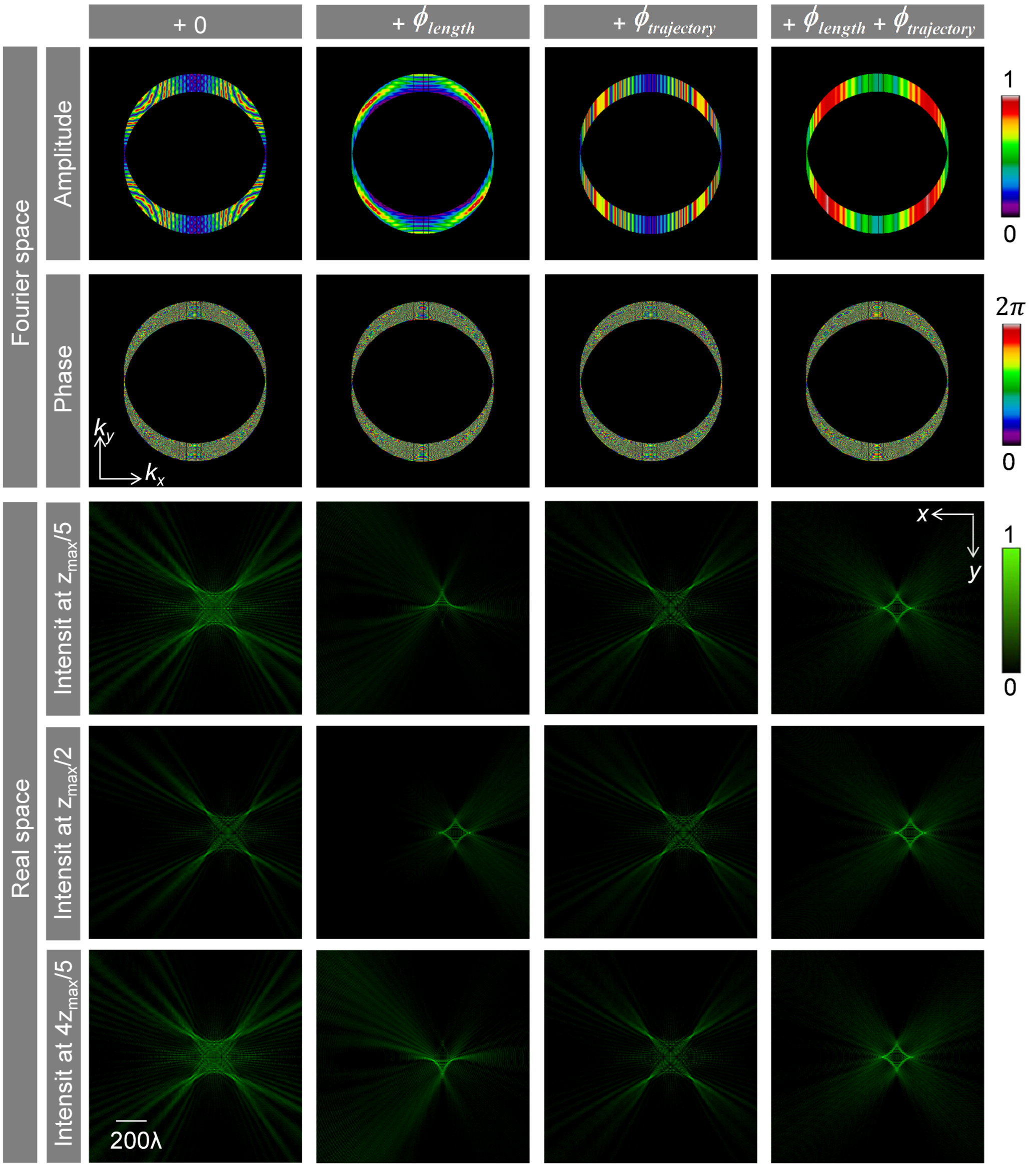

Fig. S2 Complex-amplitude distributions in Fourier space the corresponding transverse intensity distributions in real space when different compensation phases are introduced for the case depicted in Fig. 3 of the main text. Rows 1 and 2 display the amplitude and phase distributions in Fourier space, while rows 3-5 show the transverse intensity distributions at the propagation distances $z = z_{max}/5$, $z = z_{max}/2$, and $z = 4z_{max}/5$, respectively.

Comment 2: The 'OAM hidden' phenomenon is quite interesting. What's the in-depth physical mechanism to achieve this phenomenon? Maybe destructive interference?

Reply: We thank the reviewer for finding the 'hidden OAM' phenomenon interesting. In our framework, a caustic prescribed at a specific transverse plane in real space is mapped to a ring-shaped complex-amplitude distribution in Fourier space. The theoretical derivation of this correspondence is provided in Supplementary Note 3. By selectively superposing multiple Fourier-

space rings, caustics associated with different propagation planes can be independently encoded, thereby enabling a propagation-dependent evolution of OAM structures. Accordingly, the 'OAM hidden' phenomenon arises when portions of these complex-amplitude rings are intentionally omitted by setting both the amplitude and phase to zero. Therefore, the in-depth physical mechanism of this phenomenon lies in the correspondence between specific propagation planes in real space and the complex-amplitude information encoded in the Fourier-space rings, rather than destructive interference effects. To improve clarity, we have added the relevant description in the revised manuscript, as follows:

…Its intensity is spread out over a broad spatial region during propagation to conceal the beam. In our strategy, caustics at a fixed transverse plane in real space correspond to a complex-amplitude ring in Fourier space (see Supplementary Note 3, Eq. (8)). By superposing these rings, caustics at different propagation planes can be tailored independently, enabling the dynamic evolution of OAM structures of light. Furthermore, hidden OAM can be achieved when portions of the complex-amplitude rings are intentionally omitted by setting both amplitude and phase to zero. This operation suppresses the corresponding spatial-frequency components, and the optical energy is not eliminated but redistributed among the remaining ones.

Comment 3: For encryption applications, in order to achieve high security, ciphertexts and local keys are required. In the proof-of-concept demonstration, what are the ciphertexts and local keys of the decryption process?

Reply: We thank the reviewer for raising this point. The ciphertext corresponds to the caustic optical field with spatial position, beam profile, and OAM information. The local key is the lookup table (library) constructed in Fig. 5d. In the revised manuscript, we have defined them, and updated the corresponding figure captions, as follows:

To further verify the OAM encoding and its application in high-dimensional information encryption, we fabricate a metasurface composed of a 6×6 OAM beam array using TPL (see Methods and Supplementary Movie for design and fabrication details). These beams serve as ciphertexts carrying encoded information. Figure 5a presents a photograph of the fabricated sample together with SEM images in top and oblique views….

…The system is designed to sequentially match and eliminate blocks with identical structures along the propagation trajectory, analogous to the rule of the game Candy Crush. A lookup table correlating six caustic beam shapes with their corresponding average OAM is shown in Fig. 5d and acts as the decoding key for retrieving the encrypted information.

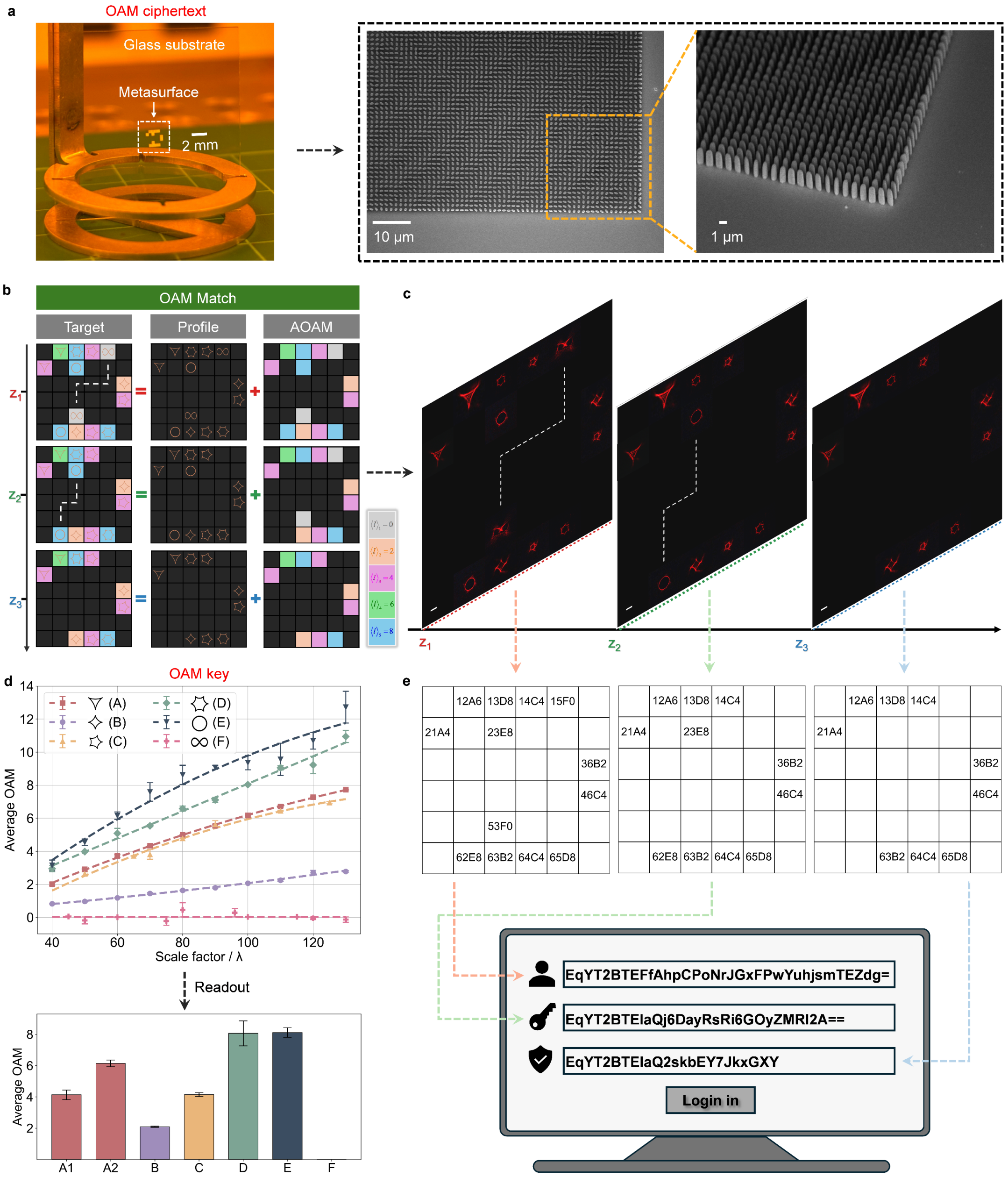

Fig. 5 Proof-of-concept experimental demonstration of optical encryption. a Photograph of the metasurface together with magnified SEM images in top and oblique views. **b** Schematic illustration of a 6 × 6 OAM beam array. The first column shows the target OAM beams at propagation distances z_1, z_2, and z_3. OAM density profiles in the second column and the average OAM in the third column are tailored as design elements. **c** Measured transverse intensity patterns at three observation planes. **d** Averaged OAM values are plotted as a function of scale factor and read out from the plot based on experimental results. **e** Decryption of the hexadecimal number combination, revealing plaintext messages including the username, password, and identification code.

Comment 4: Why did the authors characterize the average OAM value based on observing the size of intensity profiles of generated beam? Also, the authors declare that they use the OAM decomposition principle, which calculates the overlap between the light field and ideal OV modes, to calculate the OAM spectrum to obtain average OAM value. So how to measure the complex wavefront of generated light field to calculate its OAM spectrum in experiment? The authors may add some discussions and perspective on it.

Reply: We thank the reviewer for the questions and helpful suggestions. In our work, the average OAM is characterized from the transverse size of the intensity profile because the states for the generated OAM are complex mixed states rather than pure OAM eigenstates. Under such conditions, conventional OAM measurement techniques based on fork-shaped interferometric patterns become ambiguous, as the beam is no longer a single and well-defined helical phase. Importantly, we observe a strong and reproducible correlation between the average OAM and the pattern size for OAM states with nonzero OAM states. For beams exhibiting the same caustic shape, a larger average OAM leads to a larger diameter intensity profile, as shown in Fig. 5d. This monotonic relationship enables a robust and convenient means to determine the average OAM from the pattern size.

Regarding the question on calculating the OAM spectrum in experiments, we note that this could be achieved through interferometry-assisted complex-field reconstruction, as demonstrated in recent studies. Such an approach typically relies on phase-shift interference with a known reference beam, in which a probe Gaussian beam can be introduced to interfere with the generated field. By recording the intensity profiles of the measured beam, the probe Gaussian beam, and their interference fields, the complex optical field can be reconstructed, including both amplitude and phase information. Once the complex field is obtained, the OAM spectrum can be calculated by projecting the field onto ideal OAM modes according to standard OAM decomposition principles. However, a direct experimental approach to measure the OAM of our beams is still unavailable. As such, in the present work we resort to an indirect approach based on a simple and robust intensity-based characterization, which is sufficient to extract the average OAM of the generated complex states without introducing additional experimental complexity.

In the revised manuscript, we have added related discussions and perspectives in the Discussion section, as follows:

…This multidimensionality significantly increases the complexity of the encryption scheme and enhances its security against unauthorized access. The average OAM is obtained directly from the experimental scale factor in current work, but could be measured more accurately by analyzing the OAM spectrum in future work. For instance, the complex optical field of the generated beam could first be reconstructed using phase-shift interference techniques, in which the generated field interferes with a known reference beam to retrieve both amplitude and phase information[40-42]. The OAM spectrum can then be obtained by projecting the reconstructed complex field onto ideal OAM eigenmodes according to standard modal decomposition principles. This strategy has been demonstrated to provide a rigorous and quantitative characterization of OAM content for multiplexed and complex structured beams.

[41] Huang, H. et al. Phase-shift interference-based wavefront characterization for orbital angular

momentum modes. *Optics Letters* **38**, 2348-2350 (2013).
[42] Fu, S. et al. Universal orbital angular momentum spectrum analyzer for beams. *PhotoniX* **1**, 19 (2020).
[43] D'Errico, A., D'Amelio, R., Piccirillo, B., Cardano, F. & Marrucci, L. Measuring the complex orbital angular momentum spectrum and spatial mode decomposition of structured light beams. *Optica* **4**, 1350-1357 (2017).

Comment 5: The authors declare that their technique features an information capacity of I=log_2 A×B×M×P^K bits. However, there are no descriptions on the practical bound of it. For instance, how does the crosstalk between adjacent "OAM beam units" in the array limit the A×B term? What are the realistic limits for the distinguishable OAM orders (M) and beam profile classes (P)?

Reply: We thank the reviewer for raising this important question regarding the practical bounds of the information capacity. Our system operates in a sequential addressing mode, where individual units are scanned one by one. The illumination spot size is engineered to match the dimensions of a single unit, thereby spatially confining the excitation within that unit. **Consequently, inter-unit crosstalk is effectively avoided.** Nevertheless, the A×B term is not unbounded in practice and is constrained by the fabrication footprint. In our current fabrication equipment (Nanoscribe Photonic Professional GT system), the largest available substrate is a 4-inch glass wafer. Given a unit size of 375 μm × 375 μm in this work, the achievable number of addressable units is on the order of 5×10^4, which defines the upper bound of the spatial channels in our experimental configuration. With next-generation fabrication platforms such as the Nanoscribe Quantum X, which support processing of larger substrates (up to 8-inch wafers), the spatial capacity can be further increased. In addition, a smaller pixel pitch can increase the spatial sampling density and allow a larger number of independent encoding channels. However, the pixel pitch cannot be reduced arbitrarily. In diffraction-limited optical systems, the minimum meaningful pixel size is typically about $\lambda/2$, since the spatial bandwidth of the optical field is fundamentally constrained by the wavelength and further reduction will only lead to oversampling without increasing the effective spatial information capacity.

For the realistic limits of distinguishable OAM orders (M), the practical upper bound is determined by experimental distinguishability. In practice, the number of resolvable encoded OAM is limited by experimental noise, detection constraints, and measurement uncertainty. When the difference between adjacent encoded states becomes comparable to the experimental uncertainty of the measurement, their statistical distributions overlap, leading to an increased misclassification probability. Finite spatial resolution and signal-to-noise ratio limitations in the detection system further restrict the reliable discrimination of closely spaced states. Previous studies have demonstrated reliable measurements of high-order conventional OAM modes and even fractional OAM with resolutions on the order of ~0.01. However, these resolution benchmarks are established for canonical vortex beams with well-defined azimuthal phase dependence. In contrast, the OAM employed in this work is different from traditional vortex-mode formulations and exhibits non-canonical spatial phase structures. As a result, previously reported resolution limits are not applied. Determining the practical distinguishability bounds for such OAM fields requires dedicated

characterization methodologies. Techniques such as phase-shift interferometry, high-precision digital holography, or modal decomposition approaches or other advanced methods may provide viable pathways for quantifying the resolvable order spacing. We think establishing rigorous experimental discrimination criterion for these non-conventional OAM is an important direction for our future investigation.

For beam profile classes (P), the limiting mechanism is similar and depends on the balance between inter-class differences and experimental fluctuations. As the number of beam-profile categories increases, the structural differences between neighboring classes become progressively smaller, while practical variations remain, such as system drift, alignment tolerances, fabrication imperfections, and detector noise. When the difference between two classes becomes comparable to these fluctuations, their measured distributions begin to overlap, making reliable classification increasingly difficult. In this regime, the practical limit is governed by experimental distinguishability rather than theoretical encoding capacity. Continued improvements in detection hardware, signal processing, and AI-assisted analysis can enhance effective discrimination, although such advances primarily improve measurement precision rather than fundamentally changing the underlying physical encoding limit.

In the revised manuscript, we have clarified the array detection scheme and added a discussion on the practical limits of the information capacity, as follows:

…A 632 nm laser beam from an NKT supercontinuum laser is converted to be circularly polarized by a linear polarizer and a quarter-wave plate and projected onto the sample. The array is characterized using a unit-by-unit scanning strategy, such that only a single unit is illuminated at any given time, thereby spatially confining the coherent excitation and preventing simultaneous illumination of neighboring units, thus avoiding any potential interference from neighboring units.

…Our proposed scheme serves as a platform for five-dimensional optical encryption (spatial position (x, y, z), beam profile, and OAM) with an achievable information capacity of $I = \log_2 A \times B \times M \times P^K$ bits, where $A\times B$ denotes the number of beam units, M the average OAM, P the number of beam-profile classes, and K the number of observable transverse planes. It is important to note that the effective capacity is limited by practical constraints, including fabrication footprint, detection resolution, and distinguishability. The transverse spatial term $A\times B$ is determined by the substrate size and pixel pitch. The propagation parameter K depends on the achievable axial resolution. The modal term M is governed by the distinguishability of OAM modes under measurement techniques. In particular, the structured OAM states employed here necessitate specialized characterization approaches to ensure reliable discrimination. Moreover, the term P is bound by geometric separability under fabrication and detection variations. As the profile differences become comparable to the fabrication tolerance or measurement noise level, statistical overlap increases, leading to reduced classification reliability. Future efforts toward advanced signal processing and AI-assisted analysis could further enhance effective discrimination performance, thereby extending the practically accessible encoding space[39,40].

[39] Zhang, Z. et al. Structured light meets machine intelligence. eLight **5**, 26 (2025).
[40] Wu, N. et al. Intelligent nanophotonics: when machine learning sheds light. eLight **5**, 5 (2025).

Reviewer 2:

"This paper presents an innovative implementation of orbital angular momentum (OAM) sculpting in 3D space based on the optical catastrophe theory and complex-amplitude metasurfaces fabricated by TPL. The authors have demonstrated a capability to manipulate OAM profiles along pre-designed spatial trajectories. Very interestingly, they introduced a novel concept of hidden OAM to physically conceal information during propagation and reconstruct it at specific spatial coordinates for the application of enhanced optical security. In my opinion, this introduces a new concept of encryption that potentially surpasses traditional multiplexing methods.

Nevertheless, considering the authors' previous work on spatial caustics and the maturity of OAM research, the manuscript in its current form requires further supporting analysis to warrant publication in Nature Communications. Specifically, to emphasize the novelty rather than the incremental advances, I think it is essential to rigorously analyze the applicability of encryption and anti-eavesdropping features based on the caustic OAM."

We thank the reviewer for the positive assessment of the novelty and conceptual significance of our work, as well as for the insightful comments. We have carefully considered the points raised and provide detailed responses below.

Comment 1: Could the authors perform the comparative analysis of the presented work with the previous OAM sculpting to clearly show the main novelty of intrinsic OAM? For example, in the author's previous work (Nat. Communi. 2024, 15, 3719), the roatated energy flow of caustic beams also exhibited OAM. How different the purity of intrinsic component (99%) of presented work with other works including the author's previous work?

Reply: We thank the reviewer for this valuable comment. Previous literature related to OAM sculpting demonstrate structured OAM generation, including arbitrary OAM patterns at a specific transverse plane [Laser & Photonics Rev. 2022, 16, 2100451; Sci. Adv. 2022, 8, eabp8073] and doughnut-shaped OAM distributions along limited propagation trajectories [Nat. Commun. 2021, 12, 6249; Laser & Photonics Rev. 2024, 18, 2400226; Sci. Adv. 2025, 11, eadr9092]. While these approaches enable spatially structured OAM distributions, the design freedom is typically confined to an isolated focal plane or restricted propagation trajectories. Fully spatial control of OAM throughout three-dimensional space has not yet been systematically explored. Moreover, these sculpting frameworks generally do not explicitly incorporate the intrinsic–extrinsic distinction as a design consideration. In parallel, another line of research proposes the decomposition of intrinsic and extrinsic OAM, clarifying their dependence on the beam centroid and the choice of reference axis [Phys. Rev. Lett. 2002, 88, 053601]. These works primarily address the formulation and physical interpretation of angular momentum, providing important insights into the intrinsic–extrinsic distinction. However, they generally do not investigate spatial structuring of OAM distributions.

In our previous work [Nat. Commun. 2024, 15, 3719], we focus on designing the in-plane intensity distributions and propagation trajectories of spatial caustics. In that study, we report the observation of a rotational energy-flow phenomenon in such beams. However, this observation is only briefly presented in the Supplementary Information and remains at the level of

phenomenological description. No systematic investigation of the OAM properties is provided, and intrinsic and extrinsic components are neither decomposed nor quantified.

In contrast, the present manuscript addresses the problem at a fundamentally deeper level. Rather than merely reporting rotational energy flow, we establish a strategy to tailor OAM across multiple transverse planes by shaping optical caustics within the framework of catastrophe theory. Through complex-amplitude metasurfaces fabricated via two-photon polymerization, we construct caustic fields that deliberately steer the Poynting vectors, thereby enabling programmable OAM distributions. Crucially, the generated OAM can be designed to be predominantly intrinsic, which mitigates distortions arising from intrinsic–extrinsic mixing. Unlike earlier OAM sculpting studies that primarily emphasized beam shaping or trajectory control without resolving the intrinsic–extrinsic nature of the generated OAM, the present work quantitatively isolates and controls the intrinsic component. By performing systematic decomposition and demonstrating high intrinsic OAM purity, we move beyond phenomenological observations toward intrinsic OAM engineering. Leveraging this intrinsic character, we further demonstrate an optical encryption scheme. Therefore, the advancement of the present work lies not in refining a previously observed phenomenon, but in establishing a controllable platform for intrinsic OAM and validating its functionality in information encoding. We hope this clarification more clearly articulates the conceptual progression from observation to controlled generation and practical application.

Following the Reviewer's suggestion, we have enhanced our manuscript by more explicitly outlining the advantages of our study. The modifications are as follows:

…Exploiting OAM in 3D space provides larger key space for information security, which can be achieved through interference[24-26]. However, intrinsic OAM is not treated as a fundamental consideration in these sculpting frameworks. Moreover, the resulting fields are typically restricted to doughnut shapes and exhibit limited flexibility in steering along arbitrary trajectories. Hence, to realize complete spatial control and enhance the information capacity of optical encryption, the ability to manipulate OAM structures of light with high intrinsic components across multiple transverse planes is needed.

Comment 2: Considering the author's previous works on caustic sculpting, I think the concept of hidden OAM is one of the most intriguing novelties of this study for the applications of anti-eavesdropping. However, the claim that the beam is hidden simply because the intensity drops by 2 orders of magnitude is qualitative. To validate this as a robust security application, would the author quantify the randomness of the dispersed field in the OFF state? For example, evaluation of speckle contrast (or Shannon entropy if possible) could statistically prove that the information is indistinguishable from random noise and safe from recently developed computational decryption.

Reply: We sincerely thank the reviewer for the valuable comment regarding the hidden OAM. We agree that an intensity reduction alone is qualitative and may not be sufficient to substantiate a robust security claim, since weak but structured fields could still be exploited by computational recovery methods. In this context, our objective is to achieve physical-layer obfuscation and low detectability as a security-enhancing mechanism. To more rigorously substantiate this perspective, we have

incorporated a statistical characterization of the dispersed field in the OFF state to quantitatively assess its randomness and structural distinguishability. Specifically, detailed analyses of speckle contrast and Shannon entropy are provided in Supplementary Note 5. We have also modified the main text, as follows:

…The intensity profile, Poynting vector, and OAM density distribution derived from this design are displayed in Fig. 4d. Throughout the process, the OAM of light can be steered into a particular spatial profile and subsequently dispersed across the transverse plane. The structured intensity pattern evolves into a speckle-like distribution, accompanied by a two-order-of-magnitude reduction in the relative light intensity (see Supplementary Note 5 for quantitative analysis based on speckle contrast and Shannon entropy). This statistical obfuscation at the physical layer significantly reduces the feasibility of computational decryption by an eavesdropper, thereby strengthening the security[37,38].

[37] Goodman, J.W. *Statistical optics*, (John Wiley & Sons, 2015).
[38] Cover, T.M. *Elements of information theory*, (John Wiley & Sons, 1999).

Supplementary Note 5. Statistical characterization of hidden OAM.

To rigorously evaluate whether the OFF-state dispersed field is statistically indistinguishable from random noise, we perform a quantitative statistical characterization of its spatial intensity distribution. Two complementary metrics are adopted: speckle contrast, which quantifies spatial intensity fluctuations, and Shannon entropy, which measures the uncertainty and information content of the intensity distribution. Together, these metrics provide a systematic assessment of structural distinguishability and statistical randomness.

1. Speckle contrast analysis

To quantitatively evaluate the spatial intensity fluctuations and detectability of the dispersed optical field in the OFF state, we analyze the speckle contrast, defined as[1]

$$C = \frac{\sigma_I}{\langle I \rangle}. \tag{1}$$

where σ_I denotes the spatial standard deviation of the intensity distribution $I(x,y)$, and $\langle I \rangle$ represents the corresponding spatial mean intensity evaluated over a selected region of interest. It should cover the main dispersed field area to ensure sufficient spatial sampling while excluding background-dominated regions. For a fully developed speckle field with stochastic phase statistics, the intensity follows a negative exponential distribution, for which the speckle contrast theoretically approaches unity.

We calculate the speckle contrast of the hidden OAM presented in the main text. In our evaluation, the background correction is performed by subtracting the dark frame intensity I_{dark}, followed by clipping negative values to zero. To suppress noise contributions and ensure sufficient signal-to-noise ratio, only pixels satisfying $I(x,y) > k\sigma_{dark}$ (with $k = 3$ in this work) are included in the statistical evaluation, where σ_{dark} denotes the standard deviation of the dark frame.

As shown in Fig. S3a, the speckle contrast in the ON state is significantly larger than unity, reflecting the highly structured and non-stationary intensity distribution of the beam. In contrast, the OFF-state dispersed field yields speckle contrast values approaching unity, consistent with speckle-like randomness. This behavior indicates that the OFF-state field lacks discernible spatial structure and statistically resembles random speckle patterns rather than a structured beam.

2. Shannon entropy analysis

To further quantify statistical randomness, we evaluate the Shannon entropy of the intensity distribution[2]

$$H = -\sum_{i=1}^{N} p_i \log_2 p_i, \tag{2}$$

where p_i denotes the probability associated with the i-th intensity bin of the normalized intensity histogram. The entropy is further normalized by its maximum possible value $H_{max} = \log_2 N$ to enable direct comparison between different states. The normalized intensity histogram here is constructed using $N = 256$ bins, corresponding to an 8-bit discretization of the intensity range. As shown in Fig. S3b, the OFF-state dispersed field exhibits substantially higher normalized Shannon entropy than the ON-state structured beam and approaches the entropy level of numerically generated random phase fields. This increase in entropy reflects a broader and more uniform intensity probability distribution, indicating reduced predictability and diminished structural information content.

The combined results of speckle contrast (approaching unity) and high normalized Shannon entropy demonstrate that the OFF-state field exhibits statistical properties consistent with a random speckle field. It implies that the hidden OAM information is statistically obfuscated at the physical layer, significantly reducing the feasibility of computational decryption or reconstruction.

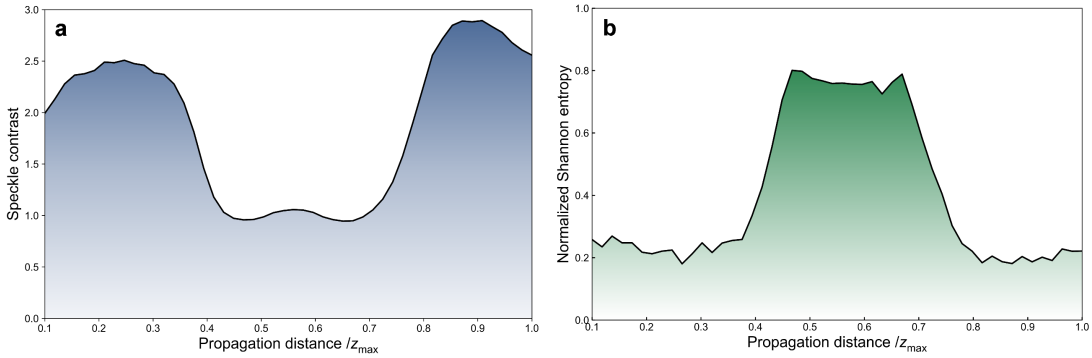

Fig. S3 Statistical characterization of the hidden OAM field. Speckle contrast (a) and normalized Shannon entropy (b) as a function of propagation distance.

[1] Goodman, J.W. *Statistical optics*, (John Wiley & Sons, 2015).

[2] Cover, T.M. *Elements of information theory*, (John Wiley & Sons, 1999).

Comment 3: Related, could the authors show the tolerance of the fabrication errors by TPL process to the hidden OAM security?

Reply: We thank the Reviewer for raising the important question. To address this concern, we have systematically evaluated the tolerance of fabrication errors introduced by the TPL process and their impact on hidden OAM security.

Fabrication errors arise from machine-related inaccuracies and can be regarded as randomly distributed geometric perturbations. In our design, the key geometric parameters include the structure height, lateral dimensions, and orientation angle. Deviations in height and lateral dimensions are treated as perturbations to the effective amplitude distribution, whereas angular misalignment is modeled as phase distortion. These fabrication imperfections are therefore represented as random amplitude and phase perturbations that reflect the magnitude of geometric deviations introduced during fabrication. In the analysis, the perturbation levels are varied within the typical tolerance range of TPL fabrication, with random amplitude and phase perturbation factors from 0 to 0.1.

To quantify their impact on hidden OAM security, we evaluate the speckle contrast and Shannon entropy of the intensity distributions across different propagation distances in both the ON and OFF states. The results indicate that, within this fabrication tolerance range, the obfuscation in the OFF state and the functionality in the ON state are both preserved.

In addition, the fabricated nanofins exhibit rounded surface features resulting from the inherent ellipsoidal focal voxel in the 3D laser printing process. In our previous study [Nat. Commun. 2024, 15, 3719], a systematic numerical analysis comparing sharp-edged and rounded geometries demonstrated that the corresponding differences in amplitude and phase responses are negligible. Therefore, the rounded profiles do not meaningfully affect the optical performance.

The detailed tolerance analysis has been added to Supplementary Note 6, and the main text has been revised accordingly, as follows:

…Leveraging on this property, the empty form can serve as a concealed encoding state, effectively hiding structural information during transmission, thereby substantially increasing the complexity of information concealment and enhancing resistance to eavesdropping and brute-force attacks. These transformations remain robust under TPL fabrication perturbations up to 10%, while statistical separability between the concealed (OFF) and constructed (ON) states is clearly preserved, demonstrating the tolerance of the hidden OAM security to fabrication imperfections (see Supplementary Note 6).

Supplementary Note 6. Robustness of hidden OAM security to fabrication imperfections.

The fabrication imperfections inherent to the TPL process can be regarded as randomly distributed geometric perturbations. These perturbations effectively reflect realistic structural deviations, including dimensional variations and rounding effects arising from the finite focal voxel. Here, we investigate their influence on the hidden OAM field. Random distribution functions f_A and f_ϕ are introduced to characterize the amplitude and phase perturbations, respectively. The corresponding random factors a and b quantify their fluctuation magnitudes. Accordingly, the perturbed amplitude and phase are expressed as $A' = A_0\left[1+af_A\right]$ and $\phi' = \phi_0\left[1+af_\phi\right]$, where A_0 and ϕ_0 denote the ideal initial amplitude and phase distributions of the hidden OAM field.

In the robustness analysis, the random factors are varied within the range of 0–0.1, corresponding to up to 10% perturbations in amplitude and phase. This interval is selected to approximate realistic fabrication tolerances in two-photon lithography. As shown in Fig. S4, the dispersed hidden OAM field in the OFF state exhibits a speckle contrast close to unity, while the normalized Shannon entropy remains within the range of approximately 0.7–0.8. These characteristics indicate that the OFF-state field is statistically indistinguishable from random noise, reflecting a high degree of statistical randomness. In contrast, in the ON state the structured OAM pattern is constructed within the designated propagation distances. The speckle contrast deviates from unity following the characteristic evolution, accompanied by a reduction in normalized Shannon entropy relative to the OFF state. Although higher perturbation levels introduce moderate spatial blurring, the statistical separation between the OFF (noise-like, high-entropy) and ON (structured, lower-entropy) regimes remains clearly preserved, ensuring reliable discrimination and robust security performance.

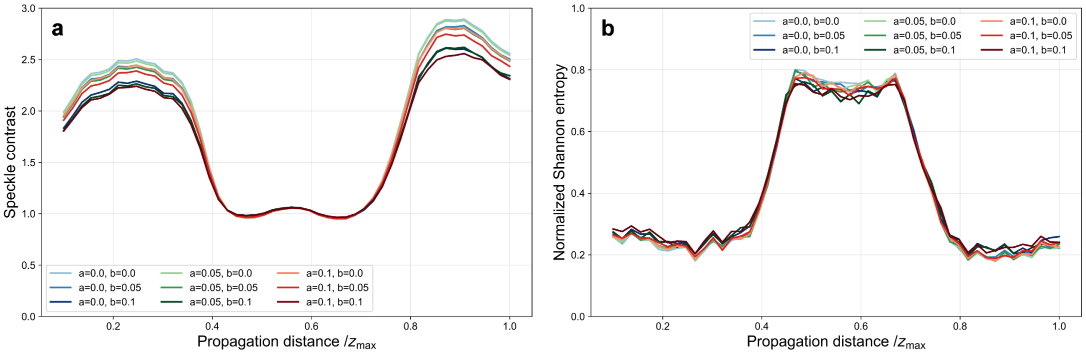

Fig. S4 Statistical characterization of the hidden OAM field under fabrication imperfections. Speckle contrast (a) and normalized Shannon entropy (b) as functions of propagation distance for varying levels of fabrication imperfections.

Comment 4: The authors demonstrate the encryption scheme using a 6 by 6 array. However, given that the period of the metasurface is larger than the wavelength, coherent diffraction such as the Talbot effect will be inevitably significant for the claimed high-capacity applications involving larger arrays (e.g., 10 by 10 or larger), which could degrade the signal by overlapping with the hidden or active OAM of neighboring region. Could the author discuss and analyze the relationship between the key parameters including pixel pitch size, propagation and capacity limit for the potential high capacity applications.

Reply: We acknowledge that when the period of the meta-atoms exceeds the wavelength, higher diffraction orders can occur. As shown in Fig. R1, the metasurface consists of meta-atoms arranged on a glass substrate with a period $P = 1.25\ \mu m$. Light propagates from the glass medium (refractive index $n_g = 1.5$) into air (refractive index $n_a = 1$). The incident and refraction angles are θ_i and θ_m, respectively. Constructive interference arises when the accumulated phase

difference between neighboring optical paths equals an integer multiple of 2π, which corresponds to an optical path difference of $m\lambda$, where m denotes the diffraction order. The diffraction criterion can be written as:

$$n_g \sin\theta_i + m\frac{\lambda}{P} = n_a \sin\theta_m.$$

Under normal illumination, only diffraction orders satisfying the propagating condition $|m\lambda / P| \le n_a$ can exist in the far field. In our configuration, this constraint limits the allowed diffraction orders to $m = 0$, ± 1, and ± 2. The corresponding diffraction angles for the first and second diffraction orders are $|\theta_{m1}| \approx 25.2°$ and $|\theta_{m2}| \approx 58.3°$, respectively.

However, the existence of higher diffraction orders does not imply the occurrence of the Talbot effect. Each unit consists of 300×300 spatially varying pixels with independently engineered nanofin geometries. As a result, the effective transmission function within a single unit lacks strict translational symmetry. The discrete phase rephasing required for the Talbot effect cannot occur at the unit level. Furthermore, at the unit array level, different units typically encode distinct complex-amplitude distributions. The array therefore lacks global translational symmetry. Since the Talbot effect fundamentally requires strict periodic repetition of identical units, this prerequisite is not satisfied in the present design. Such an effect may arise only if identical units are deliberately arranged in a strictly periodic manner.

In addition, our experiment employs a unit-by-unit scanning strategy rather than global illumination of the entire array. Because the Talbot effect relies on simultaneous coherent excitation of a strictly periodic structure, the adopted scanning scheme further suppresses the phase rephasing necessary for inter-unit self-imaging. When a single unit is illuminated, higher-order diffraction components propagate at large angles and therefore fall outside the finite entrance pupil of the microscope objective. Accordingly, only the zeroth-order beam from a single unit is collected at one time, and coherent diffraction or overlapping arising from higher-order diffraction of neighboring units is avoided.

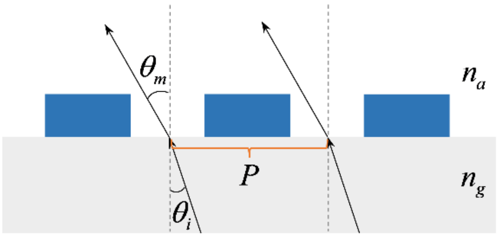

Fig. R1 Schematic cross-sectional view of the metasurface configuration.

In response to the reviewer's helpful comment, we have clarified the array detection scheme in the main text to prevent misunderstanding, as follows:

…A 632 nm laser beam from an NKT supercontinuum laser is converted to be circularly polarized by a linear polarizer and a quarter-wave plate and projected onto the sample. The array is characterized using a unit-by-unit scanning strategy, such that only a single unit is illuminated at

any given time, thereby spatially confining the coherent excitation and preventing simultaneous illumination of neighboring units, thus avoiding any potential interference from neighboring units.

Pixel pitch size to capacity limit:

In our current fabrication system (Nanoscribe Photonic Professional GT), the largest available substrate is a 4-inch glass wafer. With a pixel pitch of 1.25 μm used in this work, the achievable number of addressable pixels is on the order of 1.5×10^7. These pixels are organized into 5×10^4 units in our design, where each unit consists of 300 × 300 pixels. The number of units determines the maximum number of independently addressable spatial channels. In principle, a smaller pixel pitch can increase the spatial sampling density and allow a larger number of independent encoding channels. However, the pixel pitch cannot be reduced arbitrarily. In diffraction-limited optical systems, the minimum meaningful pixel size is typically about $\lambda / 2$, since the spatial bandwidth of the optical field is fundamentally constrained by the wavelength and further reduction will only lead to oversampling without increasing the effective spatial information capacity.

Propagation to capacity limit:

In addition to the transverse spatial degrees of freedom, propagation also plays a role in determining the achievable spatial capacity. In our design, the generated optical field exhibits diffraction-resistant propagation within a certain axial range, allowing observation at different propagation distances along the optical axis. However, the number of distinguishable observation planes is limited by the axial resolution, which depends on the effective numerical aperture of the system. In practical observations, several clear observation planes are typically selected within this propagation range.

Therefore, the achievable spatial capacity is limited by both the transverse spatial sampling defined by the pixel pitch and the number of distinguishable axial observation planes along the propagation direction. In the revised manuscript, we have added a discussion analyzing the capacity limits for potential high-capacity applications, as follows:

…Our proposed scheme serves as a platform for five-dimensional optical encryption (spatial position (x, y, z), beam profile, and OAM) with an achievable information capacity of $I = \log_2 A \times B \times M \times P^K$ bits, where $A \times B$ denotes the number of beam units, M the average OAM, P the number of beam-profile classes, and K the number of observable transverse planes. It is important to note that the effective capacity is limited by practical constraints, including fabrication footprint, detection resolution, and distinguishability. The transverse spatial term $A \times B$ is determined by the substrate size and pixel pitch. The propagation parameter K depends on the achievable axial resolution. The modal term M is governed by the distinguishability of OAM modes under measurement techniques. In particular, the structured OAM states employed here necessitate specialized characterization approaches to ensure reliable discrimination. Moreover, the term P is bound by geometric separability under fabrication and detection variations. As the profile differences become comparable to the fabrication tolerance or measurement noise level, statistical overlap increases, leading to reduced classification reliability. Future efforts toward advanced signal processing and AI-assisted analysis could further enhance effective discrimination performance, thereby extending the practically accessible encoding space[39,40].

[39] Zhang, Z. et al. Structured light meets machine intelligence. eLight **5**, 26 (2025).
[40] Wu, N. et al. Intelligent nanophotonics: when machine learning sheds light. eLight **5**, 5 (2025).

Minor points.

Comment 5: In the spectrum analysis, components below a threshold of 0.05 were removed to calculate the OAM purity. Would the authors justify this specific threshold value? A brief sensitivity analysis about the calculated purity versus different thresholds (e.g., 0.01 vs 0.1) would confirm that the reported high purity is stable and robust.

Reply: To clarify the role of the threshold, it is not used in the calculation of the OAM purity, but is introduced solely as a post-processing step to suppress experimental background noise and obtain a more reliable measured OAM spectrum. In practice, low-weight OAM components can arise from experimental imperfections such as slight beam misalignment, finite spatial sampling, and camera background noise, and are therefore not expected to represent physically meaningful OAM modes. A 5% cutoff is selected as a conservative value that lies above the typical background level while remaining well below the dominant spectral peak. Under this threshold, the dominant peak is approximately 20 times stronger than the smallest retained component, corresponding to a sufficiently large effective dynamic range. Furthermore, lowering the threshold (e.g., to 0.01) mainly introduces additional weak background contributions, whereas increasing it (e.g., to 0.1) further suppresses residual background components and may begin to over-filter weak but potentially physical signals. In all cases, the identification of the dominant OAM mode remains unchanged, indicating that the threshold primarily affects the level of background suppression and does not alter the dominant OAM content of the beam, as shown in Fig. R2. This behavior is also consistent with the effective dynamic range of measurement systems (or detectors).

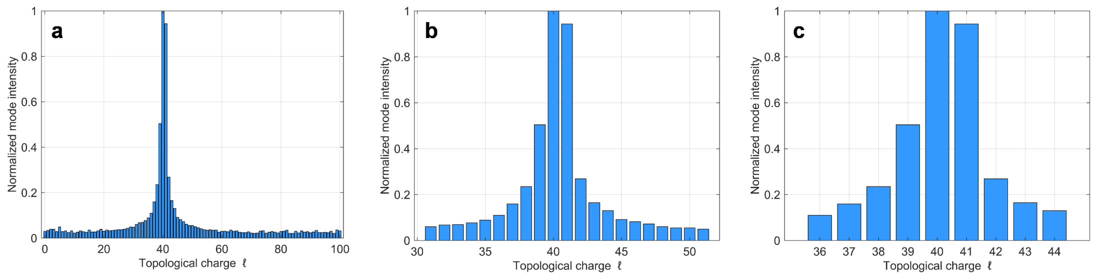

Fig. R2 OAM spectra with a 1% cut-off (a), a 5% cut-off (b), and a 10% cut-off (c), corresponding to the case shown in Fig. S1.

Accordingly, we have revised manuscript as follows:

…The correlation coefficient between the two fields can reach 99.16% in this case, which demonstrates the reliability of the OAM spectrum decomposition method. Note that the components with weights below a threshold of 0.05 can be treated as noise-dominated contributions and are therefore excluded from the analysis to suppress the influence of experimental background.

Comment 6: In Figure 5d, the scale factor serves as the lookup key to retrieve the average OAM value for decryption. While it intuitively implies the size of the pattern, an explicit definition is missing.

Reply: We thank the reviewer for the careful reading and helpful reminder. In our study, the scale factor indeed implies the size of the pattern. An explicit definition of the scale factor has now been added in the revised manuscript, as follows:

…The average OAM is determined by measuring the pattern size of the experimental patterns and retrieving the corresponding values from a database of simulated results, as shown in Fig. 5d. Here, the pattern size is characterized by the scale factor, which is used as the lookup key in the decryption process. The detailed expressions for the scale factor corresponding to different shapes are provided in Supplementary Note 8.

Supplementary Note 8. Scale factor expressions for different shapes.

The scale factor is denoted by a, which characterizes the overall size of the pattern. For all parametric equations, the parameter t is defined in the range $t \in (0, 2\pi]$.

Circle:

$$x(t) = a\cos t, \qquad y(t) = a\sin t.$$

Lemniscate of Bernoulli:

$$x(t) = \frac{\sqrt{2}a\cos t}{1+\sin^2 t}, \qquad y(t) = \frac{\sqrt{2}a\sin t\cos t}{1+\sin^2 t}.$$

n-cusped hypocycloid:

$$x(t) = (a - a/n)\cos t + a/n\cos(\frac{a-a/n}{a/n}t),$$
$$y(t) = (a - a/n)\sin t - a/n\sin(\frac{a-a/n}{a/n}t).$$

We would like to thank the reviewers again for their critical and insightful comments, which have helped us improve our manuscript. We hope that all the concerns have been adequately addressed and that the revised manuscript now fulfills the requirements for publication.
